# An Automatically Adaptive Digital Health Intervention to Decrease Opioid-Related Risk While Conserving Counselor Time: Quantitative Analysis of Treatment Decisions Based on Artificial Intelligence and Patient-Reported Risk Measures

**DOI:** 10.2196/44165

**Published:** 2023-07-11

**Authors:** John D Piette, Laura Thomas, Sean Newman, Nicolle Marinec, Joel Krauss, Jenny Chen, Zhenke Wu, Amy S B Bohnert

**Affiliations:** 1 Ann Arbor Department of Veterans Affairs Center for Clinical Management Research Ann Arbor, MI United States; 2 Department of Health Behavior Health Education, School of Public Health, University of Michigan Ann Arbor, MI United States; 3 Department of Anesthesiology, School of Medicine, University of Michigan Ann Arbor, MI United States; 4 Department of Emergency Medicine, Trinity Health St. Joseph Mercy Ann Arbor, MI United States; 5 Department of Biostatistics, School of Public Health, University of Michigan Ann Arbor, MI United States

**Keywords:** artificial intelligence, opioid safety, telehealth, reinforcement learning, pain management

## Abstract

**Background:**

Some patients prescribed opioid analgesic (OA) medications for pain experience serious side effects, including dependence, sedation, and overdose. As most patients are at low risk for OA-related harms, risk reduction interventions requiring multiple counseling sessions are impractical on a large scale.

**Objective:**

This study evaluates whether an intervention based on reinforcement learning (RL), a field of artificial intelligence, learned through experience to personalize interactions with patients with pain discharged from the emergency department (ED) and decreased self-reported OA misuse behaviors while conserving counselors’ time.

**Methods:**

We used data representing 2439 weekly interactions between a digital health intervention (“Prescription Opioid Wellness and Engagement Research in the ED” [PowerED]) and 228 patients with pain discharged from 2 EDs who reported recent opioid misuse. During each patient’s 12 weeks of intervention, PowerED used RL to select from 3 treatment options: a brief motivational message delivered via an interactive voice response (IVR) call, a longer motivational IVR call, or a live call from a counselor. The algorithm selected session types for each patient each week, with the goal of minimizing OA risk, defined in terms of a dynamic score reflecting patient reports during IVR monitoring calls. When a live counseling call was predicted to have a similar impact on future risk as an IVR message, the algorithm favored IVR to conserve counselor time. We used logit models to estimate changes in the relative frequency of each session type as PowerED gained experience. Poisson regression was used to examine the changes in self-reported OA risk scores over calendar time, controlling for the ordinal session number (1st to 12th).

**Results:**

Participants on average were 40 (SD 12.7) years of age; 66.7% (152/228) were women and 51.3% (117/228) were unemployed. Most participants (175/228, 76.8%) reported chronic pain, and 46.2% (104/225) had moderate to severe depressive symptoms. As PowerED gained experience through interactions over a period of 142 weeks, it delivered fewer live counseling sessions than brief IVR sessions (*P*=.006) and extended IVR sessions (*P*<.001). Live counseling sessions were selected 33.5% of the time in the first 5 weeks of interactions (95% CI 27.4%-39.7%) but only for 16.4% of sessions (95% CI 12.7%-20%) after 125 weeks. Controlling for each patient’s changes during the course of treatment, this adaptation of treatment-type allocation led to progressively greater improvements in self-reported OA risk scores (*P*<.001) over calendar time, as measured by the number of weeks since enrollment began. Improvement in risk behaviors over time was especially pronounced among patients with the highest risk at baseline (*P*=.02).

**Conclusions:**

The RL-supported program learned which treatment modalities worked best to improve self-reported OA risk behaviors while conserving counselors’ time. RL-supported interventions represent a scalable solution for patients with pain receiving OA prescriptions.

**Trial Registration:**

Clinicaltrials.gov NCT02990377; https://classic.clinicaltrials.gov/ct2/show/NCT02990377

## Introduction

### Background

The related public health crises of chronic pain and opioid analgesic (OA) misuse continue to challenge the United States [1,2]. Although OAs can effectively treat pain, many patients experience dependence, sedation, and overdose [3]. The number of drug overdose deaths in the United States surpassed 100,000 in 2021, and 80% of those deaths involved opioids [4]. Although nonprescribed opioids became a more predominant driver of opioid-related deaths after 2016 owing to the rapid escalation in heroin- and fentanyl-related overdoses, the number of prescription opioid–related overdoses remained essentially unchanged between 2010 and the latest data in 2020 [5]. Reducing prescription opioid misuse will likely have the downstream benefit of preventing illegal opioid use and overdose [6]. Risk factors for opioid misuse or addiction include past or current substance misuse, psychiatric disorders, younger age, and social or family environments that encourage misuse [7].

Many patients with chronic pain and risks of OA misuse can at least theoretically be identified during emergency department (ED) encounters. However, although risk reduction interventions based on motivational interviewing and cognitive behavioral therapies can be helpful [8-10], the reach and effectiveness of these interventions in EDs are limited. Resources within EDs typically do not allow for OA risk screening and intervention. Moreover, patients’ motivation for change wanes rapidly after ED discharge, and without reinforcement and assistance, many patients experience harms that might have been prevented through systematic follow-up [11].

Digital communication with patients via cell phone apps, text messaging, or interactive voice response (IVR) calls can facilitate OA risk monitoring and behavior change support for patients discharged from EDs [12-14]. Identifying strategies for using digital tools in ways that personalize evidence-based OA risk reduction services dynamically could dramatically increase the reach of these programs by more effectively targeting clinician time to patients most likely to benefit, while also giving patients greater access to care between face-to-face encounters.

In a previous randomized trial of 278 patients with musculoskeletal pain, we evaluated the effectiveness of reinforcement learning (RL), a type of artificial intelligence (AI), as a strategy for increasing the efficiency of cognitive behavioral therapy for chronic pain by automatically adjusting the type and duration of counseling sessions based on patient feedback regarding their status [15]. Compared with a standard 10-session program of clinician-delivered telephone psychotherapy, the RL-supported model resulted in noninferior improvements in pain-related disability (primary outcome) while using less than half the therapist time. At 6 months, significantly more patients in the RL-supported treatment arm had clinically meaningful improvements in pain-related disability (*P*=.01) and pain intensity (*P*=.03).

General overviews of RL have been published [16-18]. Like an experienced clinician, RL systems make probabilistic *guesses* among a limited set of potential treatment options or *action choices* and evaluate the impact of those decisions based on some dynamic measure of feedback (the *reward* in RL). Over time, RL systems systematically incorporate experience with previous action choices as well as information about the user to make progressively more educated guesses that optimize the reward function. Unlike more deterministic digital interventions that use tree-structured protocols to decide what actions to take (eg, what messages to deliver), RL systems use these repeated cycles of action choices and feedback in a dynamic, probabilistic manner to determine which actions optimize the reward score. In our previous trial of RL support for pain-related cognitive behavioral therapy [15], the RL system used daily reports from participants about their pedometer step counts and pain-related interference to calculate its reward score and make decisions about which of the 3 action choices or treatment-type options to deliver to each patient each week: a 45-minute therapist call, a 15-minute therapist call, or psychoeducation via IVR. As the RL system gained experience, it systematically adjusted the probability of each action choice to favor options that had the greatest predicted benefit, and the reward scores improved over time [19].

### Objectives

We developed the mobile “Prescription Opioid Wellness and Engagement Research in the ED” (PowerED) intervention using a similar strategy of RL plus digital patient feedback to reduce OA risks of patients with pain while targeting risk reduction counseling after ED discharge. The goal of this analysis was to determine whether PowerED provides evidence of intelligent adaptation over time. We hypothesized that as PowerED gained experience through interactions with patients, it would modify its decisions regarding the mode and intensity of patient intervention. We also hypothesized that PowerED would make more effective decisions over time, that is, over time, patient-reported OA risk indicators (the reward) would improve as the system learned what treatment-type decisions *worked best* for which patients. Given that we were particularly interested in PowerED’s ability to improve outcomes among patients with the highest risk for OA-related harms, we examined changes over time in patient-reported OA risk behaviors within strata defined by patients’ risk at enrollment (ie, baseline Current Opioid Misuse Measure [COMM] scores [20,21]).

## Methods

The methods and findings of this study are presented here in accordance with the CONSORT (Consolidated Standards of Reporting Trials)-AI Extension Guidelines [22]. All participants provided written informed consent.

### Ethics Approval

The study was approved by the University of Michigan Human Subjects Committee (#HUM00105229) before any patient contact. The protocol is registered in ClinicalTrials.gov (#NCT02990377).

### Eligibility and Recruitment

Potential participants were identified between November 8, 2018, and May 8, 2021, based on medical record evidence of visits to EDs affiliated with 2 large health systems. Both EDs are level I trauma centers located in the same county in Southeast Michigan, which has a median annual household income of US $75,730. Demographically, 70% of county residents are White non-Hispanic, 12% are Black or African American, 5% are Hispanic, and 13% are Asian or multiracial. In total, 11% of the county residents use Medicaid as their primary form of health insurance. Eligible patients were adults aged 18 to 70 years, presenting at the ED primarily for a pain-related complaint, and who reported any OA misuse in the previous 3 months. Misuse was defined as any score >0 on the modified COMM questionnaire (Multimedia Appendix 1). Patients were excluded if they were unable to provide informed consent (eg, acute intoxication or mental incompetence), were under treatment for cancer pain, met criteria for a *Diagnostic and Statistical Manual for Mental Disorders Version 5* (DSM-V) moderate or severe opioid use disorder, were experiencing opioid withdrawal symptoms at the time of screening, were unable to communicate in English, were at acute risk for self-harm, were incarcerated, were classified as level 1 trauma (eg, unconscious, intubated on respirators, or had abnormal vital signs), were pregnant, were under treatment for sexual assault, or had a family member or significant other enrolled in the study. Patients were approached for recruitment in person in the ED or were recruited via telephone following discharge because of COVID-19 restrictions. Participants were not compensated for completing IVR calls or telephone counseling sessions. Participants received US $20 to US $40 for completing surveys at baseline and follow-up time points and received US $5 for providing saliva samples at various time points (up to US $160 for completing all assessments).

### Randomization

After completing the baseline assessments, the patients were randomized with equal probability to the PowerED intervention or enhanced usual care. These analyses focused exclusively on patients randomized to the PowerED group.

### PowerED Therapeutic Content and Goals

PowerED patients participated in 12 weeks of behavioral intervention addressing OA risk and pain management. Counseling and psychoeducation were based on motivational enhancement and cognitive behavioral principles and focused on improving patients’ OA safety profile, non–OA-related strategies for coping with pain (eg, modifying dysfunctional thoughts), improving overall health (eg, relaxation), and effective communication with clinicians. Before initiating the randomized trial, the PowerED intervention content was pilot-tested and found to have positive effects on opioid misuse behaviors after discharge from the ED [23]. In additional pilot testing, we evaluated patient burden and engagement with varying frequencies of IVR-delivered OA risk assessments.

### Patient Monitoring via IVR

PowerED patients received brief (<5 minute) IVR calls up to 5 times per week, in which they responded to queries recorded in a human, female voice using their touchtone keypad. During the calls, the patients reported their use of OA prescription medications, levels of pain, and related risks. Multimedia Appendix 2 provides the complete IVR script. Data reported via these brief assessments along with baseline indicators of OA risk were used by the PowerED RL algorithm to determine the type and intensity of intervention contact the patient should receive each week and to personalize the content of those interactions. The goal was for all patients to complete IVR assessments every weekday; for 20.2% (46/228) of the participants, the call frequency was decreased at the patient’s request, owing to burden.

During IVR assessments, patients’ pain severity over the previous 24 hours was assessed using the pain Numerical Rating Scale [24]. Patients who reported taking OA pills in the previous 24 hours were asked about behavioral risk factors (eg, taking medication more frequently or at a higher dose than prescribed) using questions adapted from the Pain Medication Questionnaire [25]. Patients reporting no OA use in the previous 24 hours were asked about pain-related interference, health care encounters, use of non-OA medications, and physical activity to ensure equal call length regardless of OA use.

### Risk Reduction Counseling via the PowerED Intervention

#### Treatment Options or Action Choices

Each week for each patient, PowerED selected from three options:

1. *A brief (<5-minute) IVR call including motivational messages focused on safe OA use and healthy strategies for pain management*: During these calls, patients heard messages selected from 6 categories determined based on their most recent reports about pain severity (low, medium, or high) and OA use (yes vs no).
2. *An extended (5- to 10-minute) IVR call focused on OA-related risks and behavior change strategies*: In these calls, patients received rotating questions focused on strategies for improving their pain and overall health, their beliefs regarding opioid use, and the importance of pain management. Using the motivational enhancement principle of supporting patients’ decisional autonomy [26], patients received information about opioid safety (eg, overdose prevention). Finally, patients heard risk reduction messages tailored based on their reports of pain severity and OA use (appropriate medical uses of OAs, medical misuse, and nonmedical misuse) along with a summary of their responses.
3. *A 20-minute live telephone session with the PowerED counselor*: During these calls, counselors used motivational enhancement techniques to assess patients’ recent OA and other substance use risks, discuss risk reduction, discuss symptom management either through the patient’s current strategies or new strategies, assess the patient’s beliefs about the importance of changing behavior and their confidence in being successful, and set behavior change goals. The counselors were masters-level social workers or kinesiologists with training in motivational interviewing and cognitive behavioral therapy. Counselors participated in 8 hours of refresher training guided by a structured manual before their first patient session. Counselors were supervised biweekly by a member of the Motivational Interviewing Network of Trainers [27].

#### Session-Type Selection Using RL

Before any patient interaction, PowerED began with an equal probability of selecting each of the 3 session types. As the system delivered each session type and collected feedback about participants’ OA risk behaviors via IVR, it refined its predictions as to which session type in which situation would optimize patients’ future self-reported OA risk as reflected by the *reward score* (ie, the OA risk score, defined below). On the basis of these predictions, PowerED systematically adjusted the probability distribution across session types to favor session types with greater evidence of benefit in previous interactions. PowerED made these session-type decisions for each patient each week using data from that patient’s and other patients’ previous IVR assessment responses, baseline information (specifically, baseline COMM score, baseline pain severity, and baseline number of substances used), and the patient’s ordinal session number (ie, 1st session, 2nd session, etc). Multimedia Appendix 3 provides more details about the data used in predicting future benefits for each session type in a given context. To calculate OA risk scores, patients’ responses to each item during daily IVR monitoring calls were averaged over days during the week to increase the reliability of the score. Risk scores ranged from 1 to 7, with higher scores indicating greater OA risk. Patients received a score of “1” (the best possible score) if they reported not taking any OA pills during the week. For patients reporting some OA use during the week, their score was calculated based on the following 3 items, each of which was rated by the respondent on a scale where 1=“several times,” 2=“once or twice,” and 3=“not at all”:

OA Risk Score = 7 − [(Score_(need)_ − 1) + (Score_(friend)_ −1) + (Score_(symptoms)_ − 1)]

where each item contributed a possible 2 points and where Score_(need)_=the patient’s average response during the week to the IVR question “In the past 24 hours, how often have you needed to take your opioid pain medication more often or at a greater amount than prescribed in order to relieve your pain?”; Score_(friend)_=the patient’s average response during the week to the IVR question “In the past 24 hours, how often have you taken opioid pain medication that belonged to friends, family, or someone else?”; and Score_(symptoms)_=the patient’s average response during the week to the IVR question “In the past 24 hours, how often have you used your opioid pain medication to help with other symptoms, such as problems sleeping, being nervous or anxious, or feeling sad or stressed?”

To calculate the dynamic relationship between treatment-type decisions and expected future OA risk scores, PowerED used a nonparametric algorithm (the contextual bandit algorithm, LinUCB) [28] designed to learn quickly even with sparse data. PowerED personalized these calculations using data reflecting the *state* of the system at the time of the decision, including information from the patient’s baseline assessment (COMM score, number of substances used, and pain severity), patients’ ordinal session number (ie, 1st session to 12th session), and dynamic measures of pain intensity and pain-related interference collected during IVR assessments. Multimedia Appendix 2 provides the wording of all IVR items, and Multimedia Appendix 3 provides more details about the algorithm and calculation.

In case of ties in the expected risk scores between a counselor-delivered session and one of the IVR sessions, the system selected the IVR session to conserve counselor time. In case of ties in scores between brief and extended IVR session types, the system randomly selects between them. If the patient failed to respond to IVR assessments in the previous week, the system used data from the most recent week with available information.

### Baseline Measures and Stratification Variables

Baseline surveys included measures of participants’ sociodemographic characteristics (age, gender, race, ethnicity, employment status, and educational attainment), pain-related health status, self-reported opioid use, and depressive symptoms. Depressive symptoms were measured using the 9-item Patient Health Questionnaire [29], and for descriptive analyses, summary scores were categorized using standard cutoffs to represent no significant depressive symptoms (Patient Health Questionnaire <5), mild symptom (5-9), and moderate to severe symptoms (≥10).

Baseline OA misuse was measured using the 8-item version of the COMM (Multimedia Appendix 1) [20,21]. The 8-item COMM was optimized to minimize false positives in nonpain clinic settings and was used in our pilot test of PowerED intervention elements [23]. For the 8-item scale, questions that were not directly related to opioid misuse (largely items on mood) and an item about ED visits that was not relevant for this study were excluded. The 8-item COMM has a range of 0 to 32 and does not have validated clinical cutoffs; in these analyses, COMM scores were categorized into roughly equal-sized groups representing patients with scores of “1,” “2 to 5,” and “≥6.” Patients could report on OA use via the COMM regardless of delivery mode (ie, oral pills, injections, inhaling or *snorting*, or transdermal patches), although COMM items do not explicitly ask about each mode. The baseline survey included another measure (the “Overdose Risk Behaviors” questionnaire) that asked explicitly about nonpill OA consumption. In this sample and other studies in geographically proximate EDs, we found that the nonpill modes of OA consumption were rare. In this sample, none of the participants reported injecting OAs in the previous 6 months, and only 2 participants reported snorting OAs (1 “rarely” and 1 “sometimes”).

### Descriptive Analysis

The patients’ baseline characteristics were described for the overall sample and separately within each of the 3 subgroups defined by baseline COMM scores. We calculated the total number and percentage of attempted IVR assessments that were completed within the baseline COMM score subgroups.

### Changes in PowerED Recommendations Over Time

We hypothesized that the relative frequency of decisions resulting in the selection of each of the 3 session types would evolve as PowerED gained experience with the population and learned which options were most successful in optimizing patients’ weekly OA risk scores while conserving counselor time. To test this hypothesis, we fitted a multinomial logit model predicting the 3-level outcome of selected session type (ie, brief IVR, extended IVR, or live counseling call), controlling for the potential correlation of weekly decisions within patients. Predictors of interest included the ordinal week of enrollment (with values of 1-130) and the session number for that patient (1st session to 12th or final session). We used Stata’s (v14.2; StataCorp LLC) postestimation commands to calculate the probability distribution across session types over enrollment weeks and present those findings graphically.

### Changes in Self-Reported OA Risk Scores (Reward Scores Used by the Intervention) Over Enrollment Weeks

We hypothesized that IVR-reported OA risk scores would improve as PowerED interacted with the population and made increasingly effective decisions. To test this hypothesis, we fitted 2 nested multilevel regression models with the week’s OA risk score as the outcome and independent variables representing enrollment week, session number, and baseline OA risk severity (3-level COMM score). The session number was included to control for within-patient changes in risk scores resulting from regression to the mean or intervention effectiveness. By controlling for session number, coefficients associated with enrollment week or *calendar time* should be independent of within-patient temporal changes and represent a reasonable estimate of improvements over time in the intervention’s ability to target session types. As noted earlier, scores representing the patient’s OA risk behaviors in a given week had a possible range of 1 to 7, and 80.89% (1316/1627) of completed call-weeks had the best possible score of 1. Poisson regression was used to accommodate this skewed outcome distribution with a limited possible range. Regression models treated patients as random effects and other predictors as fixed effects. As we were particularly interested in PowerED’s performance within patient subgroups defined by baseline levels of OA risk, we first fitted a base model including only patients’ enrollment week and session number as predictors. We then fitted a second model including these predictors as well as dummy variables representing the main effects for baseline COMM scores of 2 to 5 and ≥6 (vs a score of 1) and interaction terms between those indicators and enrollment week. Using Stata’s postestimation command, we plotted the changes in predicted reward scores over enrollment weeks separately for the 3 levels of baseline COMM scores.

## Results

### Sample Description

A total of 228 patients were randomized to receive the PowerED intervention (Table 1) over 130 weeks. The average age of the participants was 40.4 years; 66.7% (152/228) of the participants were women and 64.9% (148/228) were White. Most participants (117/228, 51.3%) were employed at enrollment, and 59.6% (136/228) of the participants had at most a high school education. Most participants (175/228, 76.8%) reported chronic pain, with a mean pain intensity of 8.4 (SD 1.9) [30]. Participants with higher baseline COMM scores were more likely than those with lower scores to be Black or African American and were less likely to report a college education (*P* values for both the COMM by race and COMM by education differences were <.03). Participants with higher baseline COMM scores were also more likely to have moderate or severe symptoms of depression (*P*=.002).

**Table 1:** Baseline characteristics by baseline COMM^a^ score.

			Total (N=228)	Baseline COMM score				*P* value	
				1 (n=65)	2-5 (n=115)	≥6 (n=48)			
Age (years), mean (SD)			40.4 (12.7)	39.5 (12.8)	41.3 (13.4)	39.5 (10.6)	.57		
Women, n (%)			152 (66.7)	41 (63.1)	78 (67.8)	33 (68.8)	.76		
**Race, n (%)**									<.001
	Black		55 (24.1)	9 (13.9)	28 (24.4)	18 (37.5)			
	White		148 (64.9)	50 (76.9)	74 (64.4)	24 (50)			
	Asian		4 (1.8)	1 (1.5)	3 (2.6)	0 (0)			
	Native American		8 (3.5)	2 (3.1)	4 (3.5)	2 (4.2)			
	Multiple or other race		13 (5.7)	3 (4.6)	6 (5.2)	4 (8.3)			
Hispanic, n (%)			10 (4.6)	3 (4.9)	5 (4.6)	2 (4.4)	.93		
Unemployed, n (%)			117 (51.3)	26 (40)	58 (50.4)	33 (68.8)	.10		
**Education completed, n (%)**									.03
	Less than high school		18 (7.9)	3 (4.6)	8 (7)	7 (14.6)			
	High school		118 (51.8)	29 (44.6)	59 (51.3)	30 (62.5)			
	College or more		92 (40.4)	33 (50.8)	48 (41.7)	11 (22.9)			
COMM total score, mean (SD)			3.7 (3.4)	1.0 (0)	3.0 (1.0)	9.0 (3.6)	<.001		
Chronic pain, n (%)			175 (76.8)	43 (66.2)	89 (77.4)	43 (89.9)			
Pain intensity, mean (SD)			8.4 (1.9)	8.0 (2.5)	8.5 (1.7)	8.8 (1.3)	.05		
**Opioid prescription, n (%)**									.005
		Prescription for chronic pain	68 (29.8)	14 (21.5)	31 (27)	23 (47.9)			
		Prescription for acute pain only	46 (20.2)	10 (15.4)	29 (25.2)	7 (14.6)			
		No active prescription	84 (36.8)	27 (41.5)	40 (34.8)	17 (35.4)			
		Unknown	30 (13.2)	14 (21.5)	15 (13)	1 (2.1)			
**Depressive symptoms^b^, n (%)**									.002
	No significant symptoms (PHQ^c^-9: 0 to 4)		48 (21.3)	24 (36.9)	20 (17.9)	4 (8.3)			
	Mild symptoms (PHQ-9: 5 to 9)		73 (32.4)	17 (26.2)	41 (36.6)	15 (31.3)			
	Moderate to severe symptoms (PHQ-9: ≥10)		104 (46.2)	24 (36.9)	51 (45.5)	29 (60.4)			

^a^COMM: Current Opioid Misuse Measure.

^b^3 participants had missing data.

^c^PHQ: Patient Health Questionnaire.

### Intervention Engagement

Participants collectively received 11,544 days of IVR assessment calls and responded approximately half of the time (5891/11,544, 51.03%). Response rates were similar across the groups, as defined by the baseline COMM scores (Table 2). The RL algorithm had data from at least 1 completed IVR assessment and was able to make a decision based on that week’s information during 73.3% (2001/2730) patient-weeks of intervention engagement. Typical of IVR interventions with weekly follow-up [31], the proportion of patients completing ≥1 IVR call decreased over the 12 weeks of patients’ participation, from 89.5% (204/228) in week 1 to 65.9% (143/217) in week 12. Overall, PowerED’s RL algorithm selected brief IVR calls 42.49% (1036/2438) of the time, extended IVR calls 35.39% (863/2438) of the time, and live counseling calls 22.1% (539/2438) of the time. When patients were assigned a live call, they completed that session 58.6% (316/539) of the time, with somewhat higher completion rates (73/113, 64.6%) among patients with baseline COMM scores of ≥6.

**Table 2:** Interactive voice response (IVR) call completion, reinforcement learning (RL) decisions, and live counseling sessions completed.

			Total		Baseline COMM^a^ score				
					1		2 to 5		≥6
**Total number of IVR call-days**			11,544		3034		6083		2427
	Call-days completed, n (%)	5891 (51.03)		1662 (54.78)		3014 (49.54)		1215 (50.01)	
**Total number of IVR call-weeks**			2730		775		1370		585
	Call-weeks completed, n (%)^b^	2000 (73.26)		578 (74.58)		1004 (73.28)		418 (71.45)	
**Total number of RL decisions**			2438		671		1229		538
	Brief IVR decisions, n (%)	1036 (42.49)		289 (43.07)		496 (40.36)		251 (46.65)	
	Extended IVR decisions, n (%)	863 (35.4)		241 (35.92)		448 (36.45)		174 (32.34)	
	Live counseling decisions, n (%)	539 (22.11)		141 (21.01)		285 (23.19)		113 (21)	
Counselor sessions completed, n (%)			316 (58.63)		83 (58.86)		160 (56.14)		73 (64.60)

^a^COMM: Current Opioid Misuse Measure.

^b^Completed weeks include weeks where at least 1 call was completed.

### Changes in Treatment-Type Decisions Over Enrollment Weeks

**Figure 1:**
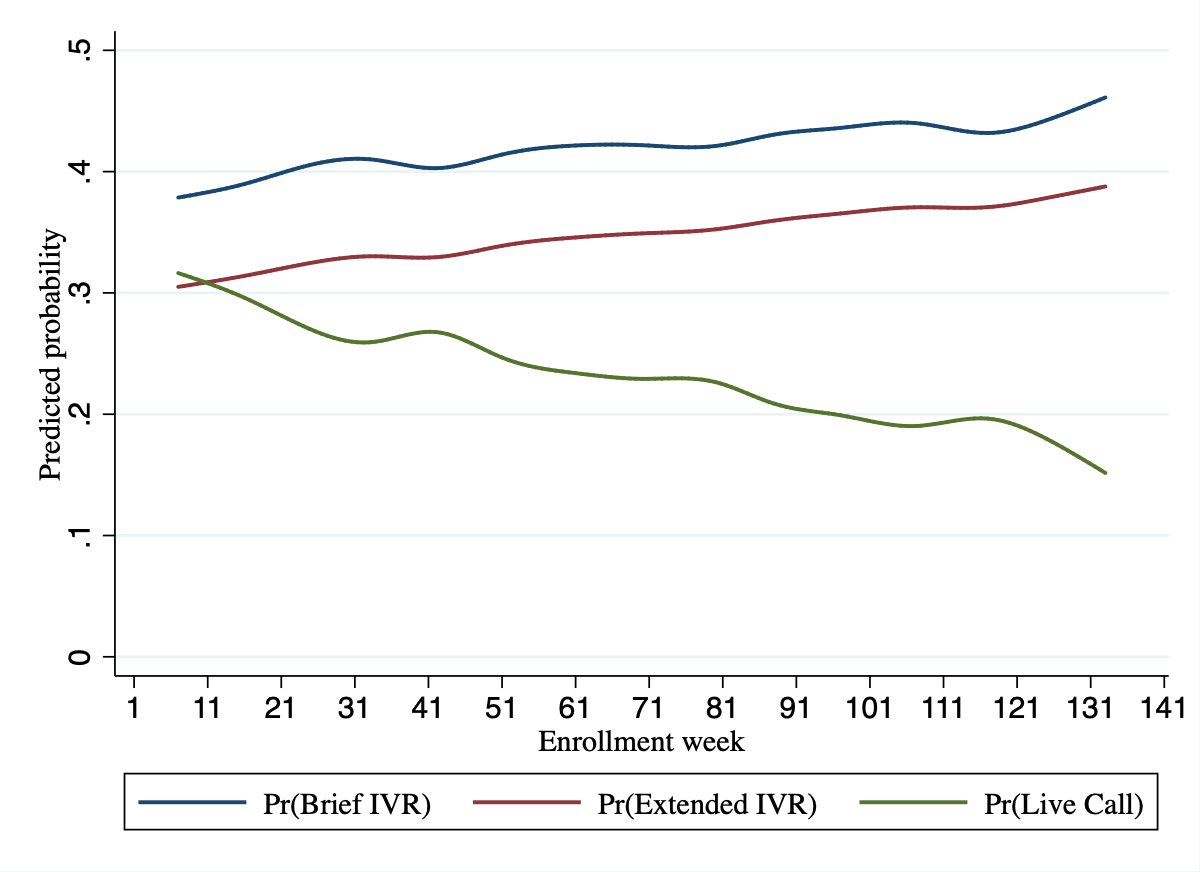
Distribution of treatment-type decisions (action choices) over time. Results were estimated using postestimation commands in Stata (v14.2) and the multinomial logit model coefficients shown in Table 3. IVR: interactive voice response; Pr: predicted probability.

PowerED’s decisions evolved as it gained experience through interactions with patients (Figure 1). Analyses predicting PowerED decisions as a function of time (enrollment week) and session number indicated that the number of live counseling sessions decreased as the intervention gained experience through patient interactions. Live counseling sessions were selected 33.5% of the time in the first 5 weeks of interactions (95% CI 27.4%-39.7%) but only for 16.4% of the sessions (95% CI 12.7%-20%) after 125 weeks. Live counseling decreased relative to brief IVR sessions (Table 3; β=−.0054; *P*=.006) and extended IVR sessions (*P*<.001).

**Table 3:** Coefficients from the multinomial logit model predicting PowerED’s^a^ recommendation of brief interactive voice response (IVR), extended IVR, or live counseling sessions.

	Extended IVR versus brief IVR			Live session versus brief IVR	
	β (95% CI)	*P* value	β (95% CI)		*P* value
Enrollment week^b^ (1 to 130)	.0059 (−.0020 to .0032)	.45	−.0054 (−.0093 to −.0016)		.006
Session number^a^ (1-12)	−.0052 (−.0353 to .0239)	.73	−.0415 (−.0818 to −.0065)		.02

^a^PowerED: Prescription Opioid Wellness and Engagement Research in the ED.

^b^Coefficient SEs were adjusted for nesting of weekly decisions within the patient.

The coefficients presented in Table 3 also suggest that, controlling for the enrollment week, PowerED learned to adjust the relative frequency of each session type depending on patients’ ordinal session number. Specifically, as patients progressed in their program, PowerED decreased the proportion of live counseling sessions relative to brief IVR sessions (Table 3; β=−.0415; *P*=.02) and extended IVR sessions (data not shown; *P*=.03). For example, after 120 weeks of interactions with the sample, PowerED chose live counseling 21.2% of the time for patients’ first session (95% CI 16.8%-25.6%) but only 13.8% of the time if the patient was in their final, week-12 session (95% CI 9.5%-18.1%).

### Changes in Patients’ OA Risk Scores Over Enrollment Weeks

**Figure 2:**
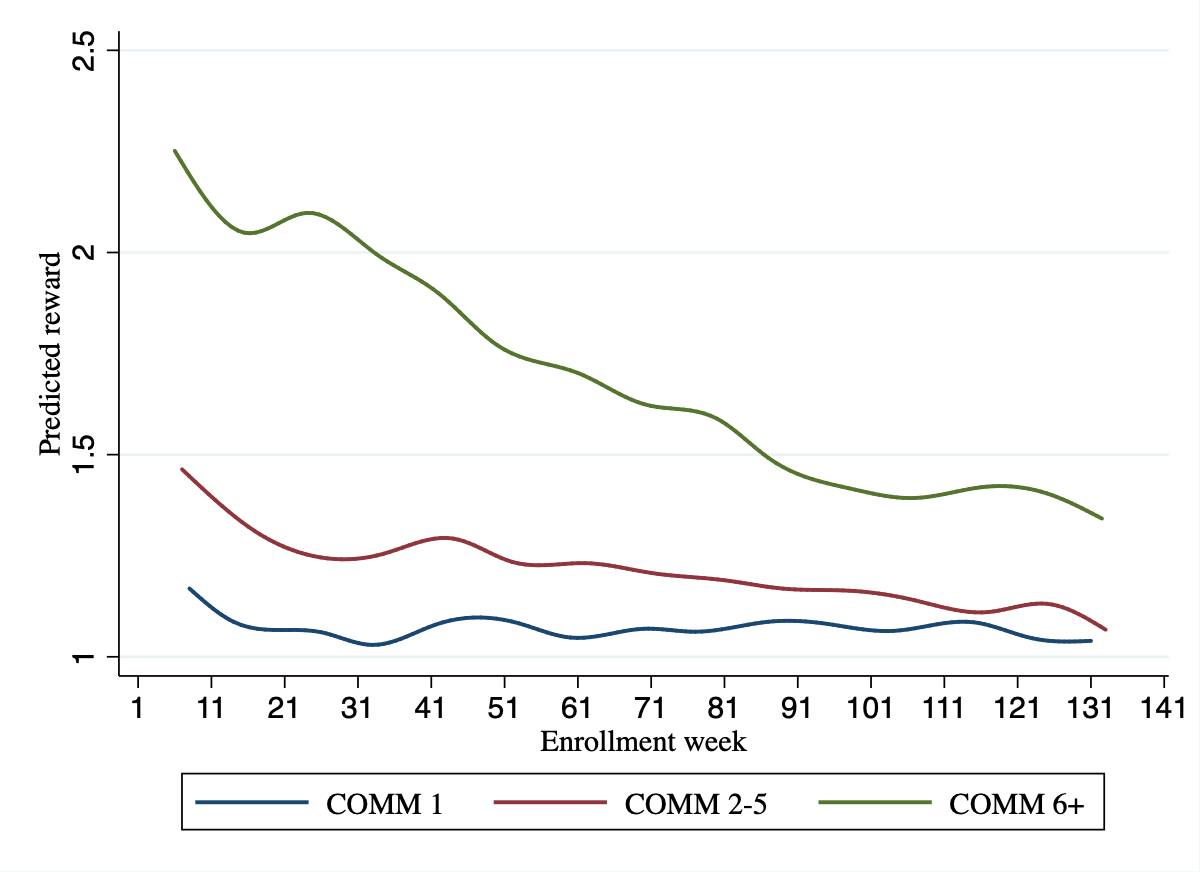
Changes in predicted opioid analgesic (OA) risk scores over enrollment weeks in groups defined by baseline Current Opioid Misuse Measure (COMM) scores, where lower scores indicate lower OA risk. Results were estimated using a multilevel model controlling for the nesting of decision weeks by patient and the coefficients shown in model 2 in Table 4. The improvement in risk scores over time for patients with baseline COMM scores of ≥6 was statistically significant (*P*=.02).

Multilevel models predicting reward scores reflecting patients’ OA risk behaviors indicated that scores improved as PowerED gained more experience through patient interactions. The model predicting scores based on both enrollment week and ordinal session number indicated that both of these 2 factors were predictive of improvements in OA risk (model 1 in Table 4; both coefficients’ *P*≤.004). Modeling also indicated that significant improvements in weekly reward scores over calendar time were experienced specifically among patients with baseline COMM scores of ≥6 indicating the highest baseline OA risk (model 2 in Table 4; *P*=.02). Using coefficients from model 2 in Table 4, risk scores among patients with baseline COMM scores of ≥6 were estimated to decrease (ie, improve) on average from 2.3 (95% CI 1.69-2.81) in week 1 of the intervention’s interaction with the target sample to 1.30 (95% CI 1.02-1.58) after 120 weeks of interactions with patients (Figure 2).

**Table 4:** Coefficients from nested models predicting weekly OA^a^ risk scores.

		Model 1		Model 2	
		β (95% CI)	*P* value	β (95% CI)	*P* value
Enrollment week		−.0024 (−.0040 to −.0008)	.003	.0000 (−.0013 to .0013)	.96
Session		−.0108 (−.0182 to −.0033)	.004	.0092 (−.0186 to −.0002)	.047
**COMM^b^ score**					
	2 to 5 (yes or no)	—^c^	—	.2190 (0.0093 to 0.4288)	.04
	≥6 (yes or no)	—	—	.7317 (0.4377 to 1.0257)	<.001
**Interactions between COMM score and enrollment week^d^**					
	(2 to 5) × week	—	—	−.0014 (−.0035 to .0007)	.18
	(≥6) × week	—	—	−.0041 (−.0075 to −.0007)	.02

^a^OA: opioid analgesic.

^b^COMM: Current Opioid Misuse Measure.

^c^Not applicable.

^d^Reference COMM score=1.

## Discussion

### Principal Findings

RL has the potential to improve the program efficiency and the number of patients that can be served by a fixed number of clinicians. In this study, we found that an RL-supported intervention for patients with pain discharged from the ED was able to effectively allocate live telephone counseling services based on real-time feedback from patients about their OA-related risk behaviors. As the program gained experience, it decreased the proportion of sessions in which the patient received live counseling and chose to provide follow-up via a mix of brief and extended IVR interactions. Despite decreasing the amount of live counseling, patient-reported risk behaviors, as reflected in the weekly risk scores used as the RL reward function, improved as the intervention gained experience.

### Strengths and Limitations

Importantly, potential increases in program benefit over time were noted in the subgroup of patients with the highest baseline level of OA-related risk. Improvements in OA risk scores were observed after controlling for within-patient trends such as regression to the mean or the intervention’s ability to change patients’ risky behaviors over the course of their treatment (ie, between the 1st and 12th session). Moreover, improvements in patients’ risk scores over time occurred during some of the most challenging months of the COVID-19 pandemic—the time when opioid deaths were increasing and access to care for many patients was restricted [4]. Services such as PowerED that can provide support at a distance represent an important resource for patients facing access barriers, especially in the context of pandemics [32]. As the COVID-19 pandemic has subsided and stricter controls on OA prescription have been implemented, at-risk patients may need a different mix of treatment types than was learned by the PowerED intervention in this study. An obvious benefit of this approach is that it can continue to adapt to changing conditions or changes in the mix of patients being served.

These findings are important because despite studies demonstrating the effectiveness of brief behavioral interventions for improving pain and decreasing OA risk [9,10,33], the growing population of patients at risk for adverse OA outcomes lacks access to treatment. At the same time, typical multisession risk reduction programs can be inefficient, providing some sessions to patients who receive little marginal benefit. Studies indicate that half of the patients receiving cognitive behavioral therapy experience a large improvement in symptoms after only 7 to 10 weeks of a 15-week treatment course [34]. The dose-response relationship is nonlinear, with much larger improvements following early sessions and smaller incremental gains later on [35]. Other studies suggest that many patients drop out of behavioral programs because they see small benefits after achieving “good enough” improvements following initial sessions [36].

As our goal was to reach the broadest possible population, this study included patients with relatively low baseline COMM scores. Having a lower threshold for inclusion in OA risk reduction could have substantial public health benefits, but monitoring and behavior change support for such a population using only live counseling sessions is not a scalable option, given the millions of eligible patients. Therefore, programs such as PowerED that seek to improve patients’ risk profile while minimizing human resource use are especially vital. It is particularly encouraging that analyses demonstrated that PowerED was able to improve patients’ self-reported risk behaviors as it gained experience interacting with patients who had COMM scores of ≥6. As shown in Table 1, such patients often have a variety of vulnerabilities and needs, including being disproportionately African American, being unemployed, having chronic pain, and having moderate to severe depressive symptoms. Among patients with lower COMM scores at baseline, we did not detect significant improvements over time, largely owing to floor effects (low baseline risk) and possible insensitivity of the weekly reported OA risk scores (ie, the reward function) at the lower extreme of the range.

In a previous study, an RL-supported program of pain cognitive behavioral therapy achieved outcomes that were noninferior to a standard therapist-delivered program [15]. Despite requiring less than half of the clinician time, more patients receiving the RL-supported program had clinically meaningful improvements in pain-related disability and pain intensity at 6 months, and simulation analyses suggested that the intervention would continue to increase its efficacy if it were exposed to a larger sample of patients [19]. These findings suggest that further research using RL-based interventions to improve opioid safety is warranted.

The PowerED intervention used patients’ IVR feedback to make decisions regarding the most effective mode of treatment while conserving counselor time. However, a limitation of this study is that response rates to IVR assessment calls were relatively low compared with the 88% response rate in a study using a similar feedback loop to adapt treatment among US Department of Veterans Affairs patients with chronic back pain [15]. Even when patients were able and willing to provide feedback, the validity and reliability of these reports were unknown, and some patients in this study likely underreported their OA-related risks. The lack of available and accurate patient feedback probably limited the ability of the intervention to effectively adapt its treatment decisions. Moreover, PowerED did not have available objective indicators of patient status, including information about patients’ medication use, overdoses, or unplanned ED visits. Future studies should consider using alternative strategies to improve the information with which adaptive programs such as PowerED make decisions, including the use of potentially more user-friendly text message monitoring [37] and direct access to clinical records. Finally, it would be valuable to evaluate the performance of this intervention in a larger sample of patients with high OA-related risk, particularly among patients from more racially and ethnically diverse communities.

This study and our previous study, in which we used a similar AI approach to deliver cognitive behavioral therapy to veterans with chronic musculoskeletal pain [15], suggest that such interventions may improve patients’ access to behavioral services while decreasing the demand for scarce counselor time. These approaches could be useful for delivering a range of complex behavioral interventions, including brief interventions addressing risky alcohol use, tobacco cessation, weight management, and mood disorders. Moreover, because these interventions personalize care automatically based on patients’ individual and group characteristics, they may provide an innovative strategy to ensure that priority populations receive the type and intensity of attention they need to achieve the best possible outcomes.

Despite these opportunities, the development and adoption of AI-supported behavioral interventions must proceed with caution and a deep understanding of the ways in which such algorithms can perpetuate or even exacerbate disparities in care. Limited diversity in AI training samples may lead to biased decisions in more heterogeneous populations [38,39]. Potential risks go beyond the statistical limitations of AI models and include the socioeconomic context in which AI-generated information about patients is used in decision-making about which patients have access to what services [40,41]. To realize the benefits of AI-supported behavioral medicine while avoiding these potential harms, experts must proceed with active collaboration among stakeholder groups representing patients and clinical teams and, most importantly, with full transparency [42,43].

### Conclusions

In conclusion, this study demonstrates that an OA risk reduction intervention supported by RL can learn to target clinician resources through interactions with patients, with the goal of maximizing population benefits. Such programs may be particularly scalable and effective for the large population of patients with pain at risk of OA-related harms who lack access to standard behavioral health services. Given that RL systems such as this one improve through experience with patients, large-scale studies in diverse patient populations are recommended to maximize intervention effectiveness and make OA risk reduction programs available for the millions of Americans at risk for OA-related harms.

## Appendix

Multimedia Appendix 1 Modified Current Opioid Misuse Measure questionnaire (used for identifying eligible participants and measuring opioid analgesic risk behavior at baseline).

Multimedia Appendix 2 Interactive voice response script.

Multimedia Appendix 3 Description of variables used in reinforcement learning decision-making.

## Abbreviations

AI: artificial intelligence
COMM: Current Opioid Misuse Measure
CONSORT: Consolidated Standards of Reporting Trials
DSM-V: Diagnostic and Statistical Manual for Mental Disorders Version 5
ED: emergency department
IVR: interactive voice response
OA: opioid analgesic
PowerED: Prescription Opioid Wellness and Engagement Research in the ED
RL: reinforcement learning

## Data Availability

Curated data sets of selected, deidentified survey data; deidentified patient responses from interactive voice response assessments; data dictionaries; and metadata from the reinforcement learning intervention will be made available on request to academic researchers via a secure data transfer. All data requests must be approved by the study’s principal investigator and appropriate human subjects and research review committees. For some requests, a signed data sharing agreement will be required. The study protocol, statistical analysis plan, and blank informed consent form will be available on request for 5 years, beginning from the date of publication.
