# Supplementary material for: An Automatically Adaptive Digital Health Intervention to Decrease Opioid-Related Risk While Conserving Counselor Time: Quantitative Analysis of Treatment Decisions Based on Artificial Intelligence and Patient-Reported Risk Measures
Source: J Med Internet Res. 2023 Jul 11;25:e44165. doi: 10.2196/44165 (PMC10369305; doi:10.2196/44165)
Supplement: Multimedia Appendix 1 [file jmir_v25i1e44165_app1.docx]

**Modified Current Opioid Misuse Measure**

Prescription pain medications, also called “opioids,” include Vicodin, codeine, Oxycontin, morphine, oxycodone, hydrocodone, methadone, hydromorphone, meperidine, fentanyl, or Norco, among others. The following questions ask about your opioid pain medication use in the **past 3 months**.

| In the **past 3 months**… | **Never** | **Rarely** | **Sometimes** | **Often** | **Very Often** |
| --- | --- | --- | --- | --- | --- |
| 1. How often have you had to go to someone other than your prescribing physician to get sufficient pain relief from opioid pain medications? (i.e., another doctor, the Emergency Room-not including today, friends, street sources) | 0 | 1 | 2 | 3 | 4 |
| 2. How often have you taken your opioid pain medications differently from how they are prescribed? | 0 | 1 | 2 | 3 | 4 |
| 3. How much of your time was spent thinking about opioid pain medications (having enough, taking them, dosing schedule, etc.)? | 0 | 1 | 2 | 3 | 4 |
| 4. How often have you needed to take opioid pain medications belonging to someone else? | 0 | 1 | 2 | 3 | 4 |
| 5. How often have you been worried about how you’re handling your opioid pain medications? | 0 | 1 | 2 | 3 | 4 |
| 6. How often have you had to take more of your opioid pain medication than prescribed? | 0 | 1 | 2 | 3 | 4 |
| 7. How often have you borrowed opioid pain medication from someone else? | 0 | 1 | 2 | 3 | 4 |
| 8. How often have you used your opioid pain medicine for symptoms other than for pain (e.g., to help you sleep, improve your mood, or relieve stress)? | 0 | 1 | 2 | 3 | 4 |
