# Supplementary material for: An Automatically Adaptive Digital Health Intervention to Decrease Opioid-Related Risk While Conserving Counselor Time: Quantitative Analysis of Treatment Decisions Based on Artificial Intelligence and Patient-Reported Risk Measures
Source: J Med Internet Res. 2023 Jul 11;25:e44165. doi: 10.2196/44165 (PMC10369305; doi:10.2196/44165)
Supplement: Multimedia Appendix 2 [file jmir_v25i1e44165_app2.docx]

IVR SCRIPT

Reducing Non-Medical Opioid Use: An Automatically Adaptive mHealth Intervention (PowerED) Study

**Principal Investigator:** Amy Bohnert, Ph.D., M.H.S.; University of Michigan School of Medicine, Department of Psychiatry, Ann Arbor, Michigan; Ann Arbor VA Center for Clinical Management Research, a Health Services Research and Development Center of Innovation, Department of Veterans Affairs, Ann Arbor, Michigan.

Email amybohne@med.umich.edu

**Co-Investigators:** Satinder Singh Baveja, Ph.D.

Frederic Blow, Ph.D.

Rebecca Cunningham, M.D.

Karen Farris, Ph.D.

Mark Ilgen, Ph.D.

John Piette, Ph.D.

Mary Janevic, Ph.D.

Joel Krauss, Ph.D.

# Outbound Validation

Note: The intro prompts below show a general overview of what the outbound intro validation section would sound like during a call.

| **INTROAnsweringMachineOrPerson**  Hello, this is a scheduled call from the University of Michigan.  [If not an answering machine, go to INTROWhoAreYou]  [If answering machine, GO TO INTROAnswerMachineDetected] |
| --- |
| **INTROAnswerMachineDetected**  Hello, this is the University of Michigan. Sorry we missed you. We will try calling back. Thank you. Good-bye.  [END CALL] |
| **INTROWhoAreYou**  We are trying to reach [Patient Name].  If this is [Patient Name], press 1.  If they are not available, press 2.  If you would like to place us on hold while you get them to the phone, press 3.  And if we reached you in error, press 4.  [IF 1, GO TO INTROEnterBirthYear]  [IF 2, GO TO INTROWillCallLater]  [IF 3, GO TO INTROHoldMusic]  [IF 4, GO TO INTROWrongNumber] |
| **INTROEnterBirthYear**  To be sure we have the right person, please enter the year you were born by pressing the numbers on the telephone keypad. For example, if you were born in 1952, please press 1 9 5 2 on your phone.  [IF VAILID BIRTH YEAR, “Thank You” and GO TO Section: Welcome]  [IF INVALID BIRTH YEAR GO TO INTROInvalidBirthYear] |
| **INTROInvalidBirthYear**  Sorry, we’re having a problem finding your information. Please re-enter your year of birth. For example, if you were born in 1952, please press 1 9 5 2 on your phone.  [If VALID BIRTH YEAR, “Thank You” and GO TO Section: Welcome]  [IF INVALID BIRTH YEAR AND TRIES < 3 GO TO INTROInvalidBirthYear]  [IF INVALID BIRTH YEAR AND TRIES >= 3 GO TO INTROValidationFailure] |
| **IntroValidationFailure**  Sorry, but we’re having a problem finding your information. To talk to someone on the study team, please call [XXX-XXX-XXXX].  [GO TO ENDCall] |
| **ENDCall**  Thank you for your time. Goodbye.  [END CALL] |
| **INTROHoldMusic**  Thank you. We will wait up to 5 minutes while you bring the participant to the phone. When you return, press any key to continue.  [PAUSE, THEN GO TO INTROPressAnyKey] |
| **INTROPressAnyKey** (append this to the end of the music file make the total 10 seconds long)  Please press any key to continue.  [ON KeyPress, “Thank You” and GO TO INTROEnterBirthYear]  [REPEAT EVERY 10 SECONDS FOR 5 MINUTES]  [ON TIMEOUT (5 MIN) END CALL] |
| **INTROWillCallLater**  Thank you for your help. We will call back at another time. Goodbye.  [END CALL] |
| **IVRInvalidResponse**  Sorry. We do not understand your response. Please try again.  [RETURN TO SENDING MODULE] |
| **INTROWrongNumber**  Sorry for bothering you. To be removed from this program, please call the study staff at [XXX-XXX-XXXX]. Thank you. Goodbye.  [END CALL] |

# Welcome

| **FROM Validation (Participant validation was successful)** |
| --- |
| **ASB_1a**  As a reminder, everything you tell us today is confidential. You may want to find a private place at this time for our call.  [GO TO ASB_1b IF CALL DAY is < 5]  [GO TO ASB_1c IF CALL DAY is > 5] |
| **ASB_1b**  *****NOTE: Only delivered during the first 3 calls*****  We’ll be asking questions about pain as well as opioid medications. These are medications which are drugs that require a prescription from a doctor and used to treat pain. Examples are Vicodin, oxycodone, hydrocodone, methadone, morphine, Percocet, and codeine. Opioids are sometimes combined with other pain relievers that are not opioids, like acetaminophen and ibuprofen. When we refer to opioid pain medications, we are talking about any medication containing an opioid.  [GO TO ASB_1c] |
| **ASB_1c**  We will start with a few questions about your experiences over the last 24 hours.  [GO TO Section: Main Questions] |

# Main Questions

| **ASB_2Q1**  On a scale of 0 to 10, with 0 being no pain at all and 10 being the worst possible pain, how would you rate your usual pain on average in the past 24 hours? Please enter the number for your rating.  [GO TO ASB_2Q2] |
| --- |
| **ASB_2Q2**  In the past 24 hours, how many opioid pills did you take? Please enter the number of pills.  [IF > 1, GO TO Section: Yes Pills Questions]  [IF 0, GO TO Section: No Pills Questions] |

# Yes Pills Questions

| **ASB_3Q1**  In the past 24 hours, how often have you needed to take your opioid pain medication more often or at a greater amount than prescribed in order to relieve your pain? If several times, press 1. If once or twice, press 2. Or if not at all, press 3.  [GO TO ASB_3Q2] |
| --- |
| **ASB_3Q2**  In the past 24 hours, how often have you taken opioid pain medication that belonged to friends, family, or someone else? If several times, press 1. If once or twice, press 2. Or if not at all, press 3.  [GO TO ASB_3Q3] |
| **ASB_3Q3**  In the past 24 hours, how often have you used your opioid pain medication to help with other symptoms, such as problems sleeping, being nervous or anxious, or feeling sad or stressed? If several times, press 1. If once or twice, press 2. Or if not at all, press 3.  [GO TO ASB_3Q4] |
| **ASB_3Q4**  In the past 24 hours, how often did pain interfere with your ability to do the things you wanted to do? If several times, press 1. If once or twice, press 2. Or if not at all, press 3.  [If Pilot, GO TO Section: Brief Message]  [If RCT, GO TO Section: RL Decision] |

# No Pills Questions

| **ASB_4Q1**  In the past 24 hours, how often did you take medications other than opioids in order to treat pain? If several times, press 1. If once or twice, press 2. Or if not at all, press 3.  [GO TO ASB_4Q2] |
| --- |
| **ASB_4Q2**  In the past 24 hours, how much time were you able to spend doing physical activity, like walking, running, playing sports, weight lifting, or yoga? If not at all, press 1. If less than [a half hour / 30 minutes], press 2. Or if [a half hour / 30 minutes] or more, press 3.  [GO TO ASB_4Q3] |
| **ASB_4Q3**  In the past 24 hours, have you been seen by a doctor or talked to a doctor or nurse by phone? If yes, press 1. If no, press 2.  [GO TO ASB_4Q4] |
| **ASB_4Q4**  In the past 24 hours, how often did pain interfere with your ability to do the things you wanted to do? If several times, press 1. If once or twice, press 2. Or if not at all, press 3.  [If Pilot, GO TO Section: Brief Message]  [If RCT, GO TO Section: RL Decision] |

# RL Decision

| **ASB_5RLDecision**  1) Brief message  2) Extended message  3) Phone session  [IF RL Engine chooses “Brief message” GO TO Section: Brief Message]  [IF RL Engine chooses “Extended message” GO TO Section: Extended Message]  [IF RL Engine chooses “Phone session” GO TO Section: Phone Session] |
| --- |

# Brief Message

| **ASB_6BM0**  Thank you for your responses.  [Play a brief message from below based on reported pain level and opioid use]  *** NOTE: Some participants may hear the same message multiple times ***  [IF ASB_2Q1 = (0,1,2) AND ASB_2Q2 = 0, GO TO Message Group 1]  [IF ASB_2Q1 = (3,4,5,6) AND ASB_2Q2 = 0, GO TO Message Group 2]  [IF ASB_2Q1 = (7,8,9,10) AND ASB_2Q2 = 0, GO TO Message Group 3]  [IF ASB_2Q1 = (0,1,2) AND ASB_2Q2 > 1, GO TO Message Group 4]  [IF ASB_2Q1 = (3,4,5,6) AND ASB_2Q2 > 1, GO TO Message Group 5]  [IF ASB_2Q1 = (7,8,9,10) AND ASB_2Q2 > 1, GO TO Message Group 6] |
| --- |
| **Brief Message Group 1: LOW PAIN (< 3), NO OPIOID USE**  **ASB_6BM_G1_1**  It sounds like you don’t have much pain right now and you did not take any opioid pain medications in the last day. It could be helpful to think about things you’ve been doing that helped you avoid having a lot of pain today.  **ASB_6BM_G1_2**  It sounds like you have found some ways to manage your pain in the last day without opioid pain medications. Using ice and heat are common ways to manage muscle and joint-pain. Ice can help with inflammation and swelling. Heat can help increase blood flow to an area to promote healing. Ice or heat should not be applied for more than 20 minutes at a time.  **ASB_6BM_G1_3**  It sounds like you don’t have much pain right now and you did not take any opioid pain medications in the past day. Some people find activity pacing to be a helpful strategy in managing pain. This means to be careful in planning how much you try to do so that you don’t push yourself too hard. For example, rather than trying to do all of a task, like washing the dishes all at once, try breaking it up into smaller periods.  **ASB_6BM_G1_4**  It sounds like you have found some ways to manage your pain in the last day without opioid pain medications. Increasing your strength through exercise can help reduce the amount of pain you experience in the future. Aim to have at least 20 minutes of exercise a day, if you don’t already meet this goal. This could be split into a couple times over the day, like a five minute walk in the morning, a ten minute walk after lunch, and another five minute walk in the evening.  **ASB_6BM_G1_5**  It sounds like you don’t have much pain right now and you did not take any opioid pain medications in the last day. Some people find meditation helpful in reducing pain, or reducing the stress that pain causes. One way to do this is called breathing from your belly. To do this, you place one hand on your chest and your other hand on your stomach while sitting or lying down. You then breathe deeply into your stomach and you will notice the hand on your stomach rising as the one on your chest falls.  **ASB_6BM_G1_6**  It sounds like you have found some ways to manage your pain in the last day without opioid pain medications. When your pain is really bothering you, try to give yourself permission to relax and do things that take your mind off of it for a while. Some people find that distractions, such as reading, watching something, listening to music, or other activities, can be helpful for taking attention away from your pain.  **ASB_6BM_G1_7**  It sounds like you don’t have much pain right now and you did not take any opioid pain medications in the past day. Sometimes people find doing meditation or relaxation techniques to be helpful in reducing pain, or reducing the stress that pain causes. As an exercise, try squeezing your right hand into a fist and holding it for five seconds. Then releasing it. Notice how the sensation changes. Next try it with your left hand. In doing this you notice that the body can hold tension and release it. You can do this progressive muscle relaxation with all parts of your body.  **ASB_6BM_G1_8**  It sounds like you have found some ways to manage your pain in the last day without opioid pain medications. Asking for support from others is another way to have your needs met so your pain does not get worse.  **ASB_6BM_G1_9**  It sounds like you don’t have much pain right now and you did not take any opioid pain medications in the past day. Some people find meditation to be helpful in reducing pain, or reducing the stress that pain causes. There are many free guided meditations available online that you could try. One meditation strategy is to close your eyes and think of relaxing each part of your body, one at a time.  **ASB_6BM_G1_10**  It sounds like you have found some ways to manage your pain in the last day without opioid pain medications. If your pain gets worse, contacting your doctor’s office may be helpful to get additional tips for managing pain. If you do not have a regular doctor, you could consider talking to friends or relatives that also experience pain, or looking up pain management options online.  **ASB_6BM_G1_11**  It sounds like you don’t have much pain right now and you did not take any opioid pain medications in the last day. Take some time to think about how you can maintain this low level of pain. What have you been doing to take care of your pain in the last couple of days?  **ASB_6BM_G1_12**  It sounds like you have found some ways to manage your pain in the last day without opioid pain medications. When people try to accomplish too much at one time, in the long run, it can cause their body to experience more pain than they would like. Try setting a timer for 15 minutes when doing household chores or yard work in order to take breaks throughout your day.  **ASB_6BM_G1_13**  It sounds like you don’t have much pain right now and you did not take any opioid pain medications in the last day. Exercise can help someone experiencing pain by loosening up stiff joints. Utilizing some of the items you have at home, like soup cans or juice containers, can be helpful substitutes for strength training weights. Try doing ten bicep curls with soup cans each day to get your muscles moving and build your strength.  **ASB_6BM_G1_14**  It sounds like you have found some ways to manage your pain in the last day without opioid medications. In addition to opioid pain medications, some people use other strategies to manage pain. Some people find breathing exercises to be helpful in reducing pain, or reducing stress and anxiety related to pain. Let’s try one of these. Start by breathing in through your nose to a count of 5. 1-2-3-4-5 Now hold the breath for a count of 5. 1-2-3-4-5 Lastly, slowly exhale through your mouth for a count of 5. 1-2-3-4-5 Repeat these a few times. This activity can be done anywhere and it is a quick way to find some relief from anxiety and stress.  **ASB_6BM_G1_15**  It sounds like you don’t have much pain right now and you did not take any opioid pain medications in the past day. Today find a time to do one of your favorite activities when you notice signs that your pain is increasing. This may help you refocus on an activity that you enjoy, and get your mind off of the pain.  **ASB_6BM_G1_16**  It sounds like you have found some ways to manage your pain in the last day without opioid pain medications. Sometimes people find doing meditation or relaxation techniques to be helpful in reducing pain, or reducing the stress that pain causes. As an exercise, try squeezing your shoulders by raising them up towards your ears. hold it for 5 seconds and then release it. Notice how relaxed your shoulders feel now. You may want to try this throughout the day, as we can hold a lot of tension in our shoulders without even noticing it.  **ASB_6BM_G1_17**  It sounds like you don’t have much pain right now and you did not take any opioid pain medications in the last day. Try asking someone to be your work out buddy. Exercise can help someone experiencing pain by loosening stiff joints, and having somebody to work out with can keep you motivated and make exercise more enjoyable.  **ASB_6BM_G1_18**  It sounds like you have found some ways to manage your pain in the last day without opioid pain medications. Getting enough sleep is important for managing pain. When your pain is bad, it might be from not getting enough sleep the night before. It could be helpful to track your sleep and look at how it affects your pain levels the next day.  **ASB_6BM_G1_19**  It sounds like you don’t have much pain right now and you did not take any opioid pain medications in the last day. Some people find meditation to be helpful in reducing pain, or reducing the stress that pain causes. There are many free guided meditations available online that you could try. One meditation strategy is to close your eyes and think about a peaceful setting. Visualize what you are smelling, seeing, hearing, and feeling in that setting.  **ASB_6BM_G1_20**  It sounds like you have found some ways to manage your pain in the last day without opioid pain medications. Making time for exercise can be challenging when you are experiencing pain. One way to incorporate exercise into your activities is to park further away from the entrance at the grocery store or using the stairs instead of the elevator.  **ASB_6BM_G1_21**  It sounds like you don’t have much pain right now and you did not take any opioid pain medications in the last day. When people try to accomplish too much at one time, in the long run, it can cause their body to experience more pain than they would like. For example, try spacing out your errands instead of trying to do them all at once.  **ASB_6BM_G1_22**  It sounds like you have found some ways to manage your pain in the last day without opioid pain medications. Ice tends to work better with new injuries by reducing inflammation and muscle spasms and pain. However, if you find relief with ice for your older injuries, continue with what you have found to be successful.  **ASB_6BM_G1_23**  It sounds like you don’t have much pain right now and you did not take any opioid pain medications in the last day. Some people find that changes in weather effect how much pain they experience. Try taking note of weather patterns and how they may overlap with your more painful days so that you may be better prepared to manage your pain.  **ASB_6BM_G1_24**  It sounds like you have found some ways to manage your pain in the last day without opioid pain medications. Staying flexible by stretching can keep you mobile longer and reduce some of the pain you are experiencing. One stretch that a lot of other people experiencing pain do is called a side stretch. While standing or sitting, lift your right arm straight above your head. Without twisting, tilt your body slowly toward your left side as far as you can go and hold it for 5 seconds. Then bring your body back to center. Lower your right arm and try it with your left arm. Reach as far to your right side as you can and hold for 5 seconds. Repeat this three times to stretch out your back and keep yourself moving each day.  **ASB_6BM_G1_25**  It sounds like you don’t have much pain right now and you did not take any opioid pain medications in the last day. In addition to opioid pain medications, some people find meditation to be helpful in reducing pain, or reducing the stress that pain causes. When you are feeling stressed out, try refocusing by making a list of things you can and cannot control. Focus on what you can control to make changes, and accept what you can’t control.  **ASB_6BM_G1_26**  It sounds like you have found some ways to manage your pain in the last day without opioid pain medications. Some people find activity pacing to be a helpful strategy in managing pain. This means to be careful in planning how much you try to do so that you don’t push yourself too hard. When doing activities like cleaning your house, try to break up the activity into manageable time. You may find it helpful to only clean one room every couple of hours so that you don’t get as tired after the activity.  **ASB_6BM_G1_27**  It sounds like you don’t have much pain right now and you did not take any opioid pain medications in the last day. People who experience pain notice that it takes a toll on their mood. Some people find it helpful to write down three things that they are grateful for each day to maintain a positive outlook on life, and change the way they experience pain. This can also help to reduce stress that pain can cause you.  **ASB_6BM_G1_28**  It sounds like you have found some ways to manage your pain in the last day without opioid pain medications. Having communication with others who experience pain can be helpful. Try joining an online group or forum for pain for comradery and pain management tips.  **ASB_6BM_G1_29**  It sounds like you don’t have much pain right now and you did not take any opioid pain medications in the last day. Heat is recommended for reoccurring injuries to increase your range of motion and improve the flexibility of tendons and ligaments. However, if you find that a combination of ice and heat works better for your pain, continue with what you found to work. It’s best not to use either, ice or heat, for more than 20 minutes at a time.  **ASB_6BM_G1_30**  It sounds like you have found some ways to manage your pain in the last day without opioid pain medications. Relaxation can be a great tool to help manage pain. Try taking an extended bath or shower. You may find it helpful to use Epsom Salt, pleasant aromas or relaxing music to help you relax even more. This may help your level of pain related stress as well as relieve some of your pain.  [After a message is played GO TO Section: Conclusion] |
| **Brief Message Group 2: MODERATE PAIN (3 - 6), NO OPIOID USE**  **ASB_6BM_G2_1**  It sounds like you have a moderate amount of pain right now, but haven’t taken any opioid pain medications. Some people find that it to be helpful to track where the pain is, how bad it is, and what you have been doing to manage the pain. That information could be helpful when discussing your pain with your doctor.  **ASB_6BM_G2_2**  It sounds like you have found some ways to manage your pain in the last day without opioid pain medications. Using ice and heat are common ways to manage muscle and joint-pain. Ice can help with inflammation and swelling. Heat can help increase blood flow to an area to promote healing. Ice or heat should not be applied for more than 20 minutes at a time.  **ASB_6BM_G2_3**  It sounds like you have a moderate amount of pain right now, but haven’t taken any opioid pain medications. Some people find activity pacing to be a helpful strategy in managing pain. This means to be careful in planning how much you try to do so that you don’t push yourself too hard. For example, rather than trying to do all of a task, like washing the dishes all at once, try breaking it up into shorter periods.  **ASB_6BM_G2_4**  It sounds like you have found some ways to manage your pain in the last day without opioid pain medications. Increasing your strength through exercise can help reduce pain over time. Aim to have at least 20 minutes of exercise a day, if you don’t already meet this goal. This could be split into a couple times over the day, like a five minute walk in the morning, a ten minute walk after lunch, and another five minute walk in the evening.  **ASB_6BM_G2_5**  It sounds like you have a moderate amount of pain right now, but haven’t taken any opioid pain medications. Some people find meditation helpful in reducing pain some, or reducing the stress that pain causes. One way to do this is called breathing from your belly. To do this, you place one hand on your chest and your other hand on your stomach while sitting or lying down. You then breathe deeply into your stomach and you will notice the hand on your stomach rising as the one on your chest falls.  **ASB_6BM_G2_6**  It sounds like you have found some ways to manage your pain in the last day without opioid pain medications. Some people find that distractions, such as reading, watching tv, listening to music, or other activities, can be helpful by taking attention away from pain.  **ASB_6BM_G2_7**  It sounds like you have a moderate amount of pain right now, but haven’t taken any opioid pain medications. Sometimes people find doing meditation or relaxation techniques to be helpful in reducing pain, or reducing the stress that pain causes. As an exercise, try squeezing your right hand into a fist and holding it for five seconds and then releasing it. Notice how the sensation changes. Next try it with your left hand. In doing this you notice that your body can hold tension and release it. You can do this progressive muscle relaxation with all parts of your body.  **ASB_6BM_G2_8**  It sounds like you have found some ways to manage your pain in the last day without opioid pain medications. Asking for support from others is another way to have your needs met so that your pain does not get worse.  **ASB_6BM_G2_9**  It sounds like you have a moderate amount of pain right now, and haven’t taken any opioid pain medication. Some people find meditation to be helpful in reducing pain, or reducing the stress that pain causes. There are many free guided meditations available online that you could try. One meditation strategy is to close your eyes and think of relaxing each part of your body, one at a time.  **ASB_6BM_G2_10**  It sounds like you have found some ways to manage your pain in the last day without opioid pain medications. Contacting your doctor’s office may be helpful to get additional tips for managing moderate pain like yours. If you do not have a regular doctor, you could consider talking to friends or relatives that also experience pain, or looking up pain management options online.  **ASB_6BM_G2_11**  It sounds like you have a moderate amount of pain right now, but haven’t taken any opioid pain medications. People who experience moderate levels of pain notice that it takes a toll on their mood. Some people find it helpful to do little acts of kindness for other people each day and write them down. This can help to maintain a positive outlook on life and reduce the stress that pain can cause you.  **ASB_6BM_G2_12**  It sounds like you have found some ways to manage your pain in the last day without opioid pain medications. When people try to accomplish too much at one time, in the long run, it can cause their body to experience more pain than they would like. Try setting a timer for 15 minutes when doing household chores or yard work in order to take breaks throughout your day.  **ASB_6BM_G2_13**  It sounds like you have a moderate amount of pain right now, and haven’t taken any opioid pain medications. Exercise can help someone experiencing pain by loosening up stiff joints. Utilizing some of the items you have at home, like soup cans or juice containers, could be helpful substitutes for strength training weights. Try doing ten bicep curls with soup cans each day to get your muscles moving and build your strength.  **ASB_6BM_G2_14**  It sounds like you have found some ways to manage your pain in the last day without opioid pain medications. In addition to opioid pain medications, some people use other strategies to manage pain. Some people find breathing exercises to be helpful in reducing pain, or reducing stress and anxiety related to pain Let’s try one of these. Start by breathing in through your nose to a count of 5. 1-2-3-4-5 Now hold the breath for a count of 5. 1-2-3-4-5 Slowly exhale through your mouth for a count of 5. 1-2-3-4-5 Repeat this a few times. This activity can be done anywhere and it is a quick way to find some relief from anxiety and stress.  **ASB_6BM_G2_15**  It sounds like you have a moderate amount of pain right now, and haven’t taken any opioid pain medications. Today find a time to do one of your favorite activities when you notice signs that pain is increasing. This may help you refocus on an activity that you enjoy, and get your mind off of the pain.  **ASB_6BM_G2_16**  It sounds like you have found some ways to manage your pain in the last day without opioid pain medications. Sometimes people find doing meditation or relaxation techniques to be helpful in reducing pain some, or reducing the stress that pain causes. As an exercise, try squeezing your shoulders by raising them up towards your ears. hold it for 5 seconds and then release it. Notice how relaxed your shoulders feel now. You may want to try this throughout the day, as we can hold a lot of tension in our shoulders without noticing it.  **ASB_6BM_G2_17**  It sounds like you have a moderate amount of pain right now, but haven’t taken any opioid pain medications. Try asking someone to be your work out buddy. Exercise can help someone experiencing pain by loosening up stiff joints, and having somebody to work out with can keep you motivated and make exercise more enjoyable.  **ASB_6BM_G2_18**  It sounds like you have found some ways to manage your pain in the last day without opioid pain medications. Getting enough sleep is important for managing pain. When your pain is bad, it might be from not getting enough sleep the night before. It could be helpful to track your sleep and look at how it affects your pain levels the next day.  **ASB_6BM_G2_19**  It sounds like you have a moderate amount of pain right now, and haven’t taken any opioid pain medications. Some people find meditation to be helpful in reducing pain, or reducing the stress that pain causes. There are many free guided meditations available online that you could try. One meditation strategy is to close your eyes and think about a peaceful setting. Visualizing what you are smelling, seeing, hearing, and feeling in that setting.  **ASB_6BM_G2_20**  It sounds like you have found some ways to manage your pain in the last day without opioid pain medications. Making time for exercise can be challenging when you are experiencing pain. One way to incorporate exercise into your activities is to park further away from the entrance at a grocery store or using the stairs instead of the elevator.  **ASB_6BM_G2_21**  It sounds like you have a moderate amount of pain right now, and haven’t taken any opioid pain medications. When people try to accomplish too much at one time, in the long run, it can cause their body to experience more pain than they would like. For example, try spacing out your errands instead of trying to do them all at once.  **ASB_6BM_G2_22**  It sounds like you have found some ways to manage your pain in the last day without opioid pain medications. Ice tends to work better for new injuries by reducing inflammation and muscle spasms and pain. Though, if you find relief with ice for your older injuries, continue with what you have found to be successful.  **ASB_6BM_G2_23**  It sounds like you have a moderate amount of pain right now, but haven’t taken any opioid pain medications. Some people find that changes in the weather effect how much pain they experience. Try taking note of weather patterns and how they may overlap with your more painful days so that you may be better prepared to manage your pain.  **ASB_6BM_G2_24**  It sounds like you have found some ways to manage your pain in the last day without opioid pain medications. Staying flexible by stretching can keep you mobile longer and reduce some of the pain you are experiencing. One stretch that a lot of other people experiencing pain do is called a side stretch. While standing or sitting, lift your right arm straight above your head. Without twisting, tilt your body slowly toward your left side as far as you can go and hold it for 5 seconds. Then bring your body back to center. Lower your right arm and try it with your left arm. Reach as far to your right side as you can and hold for 5 seconds. Repeat this three times to stretch out your back and get yourself moving each day.  **ASB_6BM_G2_25**  It sounds like you have a moderate amount of pain right now, and haven’t taken any opioid pain medications. In addition to opioid pain medications, some people find meditation to be helpful in reducing pain some, or reducing the stress that pain causes. When you are feeling stressed out, try refocusing by making a list of things you can and can’t control. Focus on what you can control to make changes, and accept what you cannot.  **ASB_6BM_G2_26**  It sounds like you have found some ways to manage your pain in the last day without opioid pain medications. Some people find activity pacing to be a helpful strategy in managing pain. This means to be careful in planning how much you try to do so that you don’t push yourself too hard. When doing activities like cleaning your house, try to break up the activity into manageable time. You may find it helpful to only clean one room every couple of hours so that you don’t get as tired after the activity.  **ASB_6BM_G2_27**  It sounds like you have a moderate amount of pain right now, and haven’t taken any opioid pain medications. People who experience moderate levels of pain notice that it takes a toll on their mood. Some people find it helpful to write down three things that they are grateful for each day to maintain a positive outlook on life, and change the way they experience pain. This can also help to reduce the stress that pain may cause you.  **ASB_6BM_G2_28**  It sounds like you have found some ways to manage your pain in the last day without opioid pain medications. Having communication with others who also experience pain can be helpful. Try joining an online group or forum for pain for comradery and pain management tips.  **ASB_6BM_G2_29**  It sounds like you have a moderate amount of pain right now, and haven’t taken any opioid pain medications. Heat is recommended for reoccurring injuries to increase your range of motion and improve the flexibility of tendons and ligaments. However, if you find that a combination of ice and heat works better for your pain, continue with what you have found to work. It’s best not to use either, ice or heat, for more than 20 minutes at a time.  **ASB_6BM_G2_30**  It sounds like you have found some ways to manage your pain in the last day without opioid pain medications. Relaxation can be a great tool to help manage pain. Try taking an extended bath or shower. You may find it helpful to use Epsom Salt, pleasant aromas or calming music to help you relax even more. This can help your level of pain related stress as well as reduce some of your pain.  [After a message is played GO TO Section: Conclusion] |
| **Brief Message Group 3: HIGH PAIN (> 6), NO OPIOID USE**  **ASB_6BM_G3_1**  It sounds like you have been in a lot of pain over the last day, but haven’t taken any opioid pain medications. Some people find that it can be helpful to track where the pain is, how bad it is, and what you have been doing to manage the pain. That information could be helpful when discussing your pain with your doctor.  **ASB_6BM_G3_2**  It sounds like you have found some ways to manage your strong pain in the last day without opioid pain medications. Using ice and heat are common ways to manage muscle and joint-pain. Ice can help with inflammation and swelling. Heat can help increase blood flow to an area to promote healing. Ice or heat should not be applied for more than 20 minutes at a time.  **ASB_6BM_G3_3**  It sounds like you have been in a lot of pain over the last day, but haven’t taken any opioid pain medications. Some people find activity pacing to be a helpful strategy in managing pain. This means to be careful in planning how much you try to do so that you don’t push yourself too hard. For example, rather than trying to do all of a task, like washing the dishes all at once, try breaking it up into shorter periods.  **ASB_6BM_G3_4**  It sounds like you have found some ways to manage your strong pain in the last day without opioid pain medications. Increasing your strength through exercise can help reduce pain over time. Aim to have at least 20 minutes of exercise a day, if you don’t already meet this goal. This could be split into a couple times over the day, like a five minute walk in the morning, a ten minute walk after lunch, and another five minute walk in the evening.  **ASB_6BM_G3_5**  It sounds like you have been in a lot of pain over the last day, but haven’t taken any opioid pain medications. Some people find meditation helpful in reducing pain, or reducing the stress that pain causes. One way to do this is called breathing from your belly. To do this, you place one hand on your chest and your other hand on your stomach while sitting or lying down. You then breathe deeply into your stomach and you will notice the hand on your stomach rising as the one on your chest falls.  **ASB_6BM_G3_6**  It sounds like you have found some ways to manage your strong pain in the last day without opioid pain medications. Some people find that distractions, such as reading, watching tv, listening to music, or other activities, can be helpful for taking attention away from pain.  **ASB_6BM_G3_7**  It sounds like you have been in a lot of pain over the last day, and haven’t taken any opioid pain medications. Sometimes people find doing meditation or relaxation techniques to be helpful in reducing pain some, or reducing the stress that pain causes, even if only by a little bit. As an exercise, try squeezing your right hand into a fist and holding it for five seconds and then releasing. Notice how the sensation changes. Next try it with your left hand. In doing this you notice that the body can hold tension and release it. You can do this progressive muscle relaxation with all muscle groups of your body.  **ASB_6BM_G3_8**  It sounds like you have found some ways to manage your strong pain in the last day without opioid pain medications. Asking for support from others is another way to have your needs met so your pain does not get worse.  **ASB_6BM_G3_9**  It sounds like you have been in a lot of pain over the last day, and haven’t taken any opioid pain medications. Some people find meditation to be helpful in reducing pain, or reducing the stress that pain causes, even if only by a little. There are many free guided meditations available online that you could try. One meditation strategy is to close your eyes and think of relaxing each or your body parts, one at a time.  **ASB_6BM_G3_10**  It sounds like you have found some ways to manage your strong pain in the last day without opioid pain medications. When pain becomes very high, it may be hard to manage alone. Contacting your doctor’s office might help provide you with options for managing strong pain like yours. If you do not have a regular doctor, you could consider talking to friends or relatives that also experience pain, or looking up pain management options online.  **ASB_6BM_G3_11**  It sounds like you have been in a lot of pain over the last day, but haven’t taken any opioid pain medications. People who experience high levels of pain notice that it takes a toll on their mood. Some people find it helpful to do little acts of kindness for other people each day and write them down. This can help to maintain a positive outlook on life and reduce the stress that pain can cause you.  **ASB_6BM_G3_12**  It sounds like you have found some ways to manage your strong pain in the last day without opioid pain medication. When people try to accomplish too much at one time, in the long run, it can cause their body to experience more pain than they would like. Try setting a timer for 15 minutes when doing household chores or yard work in order to take breaks throughout your day.  **ASB_6BM_G3_13**  It sounds like you have been in a lot of pain over the last day, but haven’t taken any opioid pain medications. Exercise can help someone experiencing pain by loosening up stiff joints. Utilizing some of the items you have at home, like soup cans or juice containers, could be helpful substitutes for strength training weights. Try doing ten bicep curls with soup cans each day to get your muscles moving and build your strength.  **ASB_6BM_G3_14**  It sounds like you have found some ways to manage your strong pain in the last day without opioid pain medications. In addition to opioid pain medications, some people use other strategies to manage pain. Some people find breathing exercises to be helpful in reducing pain, or reducing stress and anxiety related to pain. Let’s try one of these. Start by breathing in through your nose to a count of 5. 1-2-3-4-5 Now hold the breath for a count of 5. 1-2-3-4-5 Slowly exhale through your mouth for a count of 5. 1-2-3-4-5 Repeat this a few times. This activity can be done anywhere and it is a quick way to find some relief from anxiety and stress.  **ASB_6BM_G3_15**  It sounds like you have been in a lot of pain over the last day, and haven’t taken any opioid pain medications. Today find a time to do one of your favorite activities when you notice signs that pain is increasing. This may help you refocus on an activity that you enjoy, and get your mind off of the pain.  **ASB_6BM_G3_16**  It sounds like you have found some ways to manage your strong pain in the last day without opioid pain medications. Sometimes people find meditation or relaxation techniques to be helpful in reducing pain, or reducing the stress that pain causes. As an exercise, try squeezing your shoulders by raising them up towards your ears. hold it for 5 seconds and release it. Notice how relaxed your shoulders feel now. You may want to try this throughout the day, as we can hold a lot of tension in our shoulders without noticing it.  **ASB_6BM_G3_17**  It sounds like you have been in a lot of pain over the last day, but haven’t taken any opioid pain medications. Try asking someone to be your work out buddy. Exercise can help someone experiencing pain by loosening up stiff joints, and having somebody to work out with can help keep you motivated and make exercise more enjoyable.  **ASB_6BM_G3_18**  It sounds like you have found some ways to manage your strong pain in the last day without opioid pain medications. Getting enough sleep is important for managing pain. When your pain is bad, it might be from not getting enough sleep the night before. It could be helpful to track your sleep and look at how it affects your pain levels the next day.  **ASB_6BM_G3_19**  It sounds like you have been in a lot of pain over the last day, and haven’t taken any opioid pain medications. Some people find meditation to be helpful in reducing pain, or reducing the stress that pain causes. There are many free guided meditations available online that you could try. One meditation strategy is to close your eyes and think about a peaceful setting. Visualize what you are smelling, seeing, hearing, and feeling in that setting.  **ASB_6BM_G3_20**  It sounds like you have found some ways to manage your strong pain in the last day without opioid pain medication. Making time for exercise can be challenging when you are experiencing pain. One way to incorporate exercise into your activities is to park further away from the entrance at the grocery store or using the stairs instead of the elevator.  **ASB_6BM_G3_21**  It sounds like you have been experiencing strong pain in the last day without opioid pain medication. When people try to accomplish too much at one time, in the long run, it can cause their body to experience more pain than they would like. For example, try spacing out your errands instead of trying to do them all at once.  **ASB_6BM_G3_22**  It sounds like you have been in a lot of pain over the last day, and haven’t taken any opioid pain medications. Ice tends to work better for new injuries by reducing inflammation,muscle spasms, and pain. However, if you find relief with ice for your older injuries, continue with what you have found to be successful.  **ASB_6BM_G3_23**  It sounds like you have been experiencing strong pain in the last day without opioid pain medications. Some people find that changes in the weather effect how much pain they experience. Try taking note of weather patterns and how they may overlap with your more painful days so that you may be better prepared to manage your pain.  **ASB_6BM_G3_24**  It sounds like you have been dealing with some intense pain within the last day but did not take any opioid pain medications. Staying flexible by stretching can keep you mobile longer and reduce some of the pain you are experiencing. One stretch that a lot of other people experiencing pain do is a side stretch. While standing or sitting, lift your right arm straight above your head. Without twisting, tilt your body slowly toward your left side as far as you can go and hold it for 5 seconds. Then bring your body back to center. Lower your right arm and try it again with your left arm. Reach as far to your right side as you can and hold for 5 seconds. Repeat this three times to stretch out your back and get yourself moving each day.  **ASB_6BM_G3_25**  It seems you have had strong pain in the last day and have not taken any opioid pain medications. In addition to opioid pain medication, some people find meditation to be helpful in reducing pain, or reducing the stress that pain causes. When you are feeling stressed out, try refocusing by making a list of things you can and can’t control. Focus on the things you can control to make change, and accept the things that you cannot.  **ASB_6BM_G3_26**  It sounds like you have been experiencing strong pain in the last day without opioid pain medication. Some people find activity pacing to be a helpful strategy in managing pain. This means to be careful in planning how much you try to do so that you don’t push yourself too hard. When doing activities like cleaning your house, try to break up the activity into manageable time. You may find it helpful to only clean one room every couple of hours so that you don’t get as tired after the activity.  **ASB_6BM_G3_27**  It sounds like you are having significant pain but you didn’t take any opioid pain medications in the last day. People who experience high levels of pain notice that it takes a toll on their mood. Some people find it helpful to write down three things that they are grateful for each day to maintain a positive outlook on life, and change the way they experience pain. This can also be help to reduce the stress that pain can cause you.  **ASB_6BM_G3_28**  It sounds like you have been in a lot of pain over the last day, but haven’t taken any opioid pain medications. Having communication with others who also experience pain can be helpful. Try joining an online group or forum for pain for comradery and pain management tips.  **ASB_6BM_G3_29**  It sounds like you have had strong pain in the last day and haven’t used opioid pain medications. Heat is recommended for reoccurring injuries to increase your range of motion and improve the flexibility of tendons and ligaments. However, if you find that a combination of ice and heat works better for your pain, continue with what you found to work. It’s best not to use either, ice/heat, for more than 20 minutes at a time.  **ASB_6BM_G3_30**  It sounds like you have been in a lot of pain over the last day, but haven’t taken any opioid pain medications. Relaxation can be a great tool to help manage pain. Try taking an extended bath or shower. You may find it helpful to use Epsom Salt, pleasant aromas or calming music to help you relax even more. This can help with your pain related stress as well as relieve some of your pain.  [After a message is played GO TO Section: Conclusion] |
| **Brief Message Group 4: LOW PAIN (< 3), OPIOID USE**  **ASB_6BM_G4_1**  It sounds like you don’t have much pain right now. In addition to opioid pain medications, some people use other strategies to manage pain. It could be helpful to think about things you’ve been doing that helped you avoid having a lot of pain today or in the past.  **ASB_6BM_G4_2**  It sounds like your pain has been fairly under control. In addition to opioid pain medications, some people use other strategies to manage pain. Using ice and heat are common ways to manage muscle and joint-pain. Ice can help with inflammation and swelling. Heat can help increase blood flow to an area to promote healing. Ice or heat should not be applied for more than 20 minutes at a time.  **ASB_6BM_G4_3**  It sounds like you don’t have much pain right now. In addition to opioid pain medications, some people use other strategies to manage pain. Some people find activity pacing to be a helpful strategy in managing pain. This means to be careful in planning how much you try to do so that you don’t push yourself too hard. For example, rather than trying to do all of a task, like washing the dishes all at once, try breaking it up into shorter periods.  **ASB_6BM_G4_4**  It sounds like your pain has been fairly under control. In addition to opioid pain medications, some people use other strategies to manage pain. Increasing your strength through exercise can help reduce the amount of pain you experience in the future. Aim to have at least 20 minutes of exercise a day, if you don’t already meet this goal. This could be split into a couple times over the day, like a five minute walk in the morning, a ten minute walk after lunch, and another five minute walk in the evening.  **ASB_6BM_G4_5**  It sounds like you don’t have much pain right now. In addition to opioid pain medications, some people use other strategies to manage pain. Some people find meditation helpful in reducing pain some, or reducing the stress that pain causes. One way to do this is called breathing from your belly. To do this, you place one hand on your chest and the other hand on your stomach while sitting or lying down. You then breathe deeply into your stomach and you’ll notice the hand on your stomach rising as the one on your chest falls.  **ASB_6BM_G4_6**  It sounds like your pain has been fairly under control. In addition to opioid pain medications, some people use other strategies to manage pain. Some people find that distractions, such as reading, watching something, listening to music, or other activities, can be helpful for taking attention away from pain.  **ASB_6BM_G4_7**  It sounds like you don’t have much pain right now. In addition to opioid medications, some people use other strategies to manage pain. Sometimes people find doing meditation or relaxation techniques to be helpful in reducing pain, or reducing the stress that pain causes. As an exercise, try squeezing your right hand into a fist and holding it for five seconds. Then releasing it. Notice how the sensation changes. Next try it with your left hand. In doing this you notice that the body can hold tension and release it. You can do this progressive muscle relaxation with all muscle groups of your body.  **ASB_6BM_G4_8**  It sounds like your pain has been fairly under control. In addition to opioid pain medications, some people use other strategies to manage pain. Asking for support from others is another way to have your physical and emotional needs met so your pain does not get worse.  **ASB_6BM_G4_9**  It sounds like you don’t have much pain right now. In addition to opioid pain medications, some people use other strategies to manage pain. Some people find meditation to be helpful in reducing pain, or reducing the stress that pain causes. There are many free guided meditations available online that you could try. One meditation strategy is to close your eyes and think of relaxing each part of your body, one at a time.  **ASB_6BM_G4_10**  It sounds like your pain has been fairly under control. If you find that you are able to decrease opioid medication use, talking with your doctor about this may be helpful. If someone is taking opioids regularly even if only for a couple of weeks, stopping the medication can cause painful withdrawal symptoms that can be confused with the pain problem returning. A doctor can provide recommendations to lessen these symptoms.  **ASB_6BM_G4_11**  It sounds like you don’t have much pain right now. In addition to opioid pain medications, some people use other strategies to manage pain. People who experience pain notice that it takes a toll on their mood. Some people find it helpful to do little acts of kindness for other people each day and write them down. This can help to maintain a positive outlook on life and reduce the stress that pain can cause you.  **ASB_6BM_G4_12**  It sounds like your pain has been fairly under control. When people try to accomplish too much at one time, in the long run, it can cause their body to experience more pain than they would like. Try setting a timer for 15 minutes when doing household chores or yard work in order to take breaks throughout your day.  **ASB_6BM_G4_13**  It sounds like you don’t have much pain right now. In addition to opioid pain medications, exercise can help someone experiencing pain by loosening up stiff joints. Utilizing some of the items you have at home, like soup cans or juice containers, could be helpful substitutes for strength training weights. Try doing ten bicep curls with soup cans each day to get your muscles moving and build your strength.  **ASB_6BM_G4_14**  It sounds like your pain has been fairly under control. In addition to opioid pain medication, some people find breathing exercises to be helpful in reducing pain, or reducing stress and anxiety related to pain. Let’s try one of these, Start by breathing in through your nose to a count of 5. 1-2-3-4-5 Now hold the breath for a count of 5. 1-2-3-4-5 Lastly, slowly exhale through your mouth for a count of 5. 1-2-3-4-5 Repeat this a few times. This activity can be done anywhere and it is a quick way to find some relief from anxiety and stress.  **ASB_6BM_G4_15**  It sounds like you don’t have much pain right now. In addition to opioid pain medications, some people use other strategies to manage pain. Today find a time to do one of your favorite activities when you notice signs that pain is increasing. This may help you refocus on an activity that you enjoy, and get your mind off of the pain.  **ASB_6BM_G4_16**  It sounds like your pain has been fairly under control. In addition to opioid pain medications, sometimes people find meditation or relaxation techniques to be helpful in reducing pain, or reducing the stress that pain causes. As an exercise, try squeezing your shoulders by pulling them up towards your ears. Hold this for 5 seconds and then release it. Notice how relaxed your shoulders feel now. You may want to try this throughout the day, as we can hold a lot of tension in our shoulders without noticing it.  **ASB_6BM_G4_17**  It sounds like you don’t have much pain right now. In addition to opioid pain medications, some people use other strategies to manage pain. Try asking someone to be your work out buddy. Exercise can help someone experiencing pain by loosening up stiff joints, and having somebody to work out with can keep you motivated and make exercise more enjoyable.  **ASB_6BM_G4_18**  It sounds like your pain has been fairly under control. In addition to opioid pain medications, some people find getting enough sleep as an important factor for managing pain. When your pain is bad, it might be from not getting enough sleep the night before. It could be helpful to track your sleep and see how it affects your pain levels the next day.  **ASB_6BM_G4_19**  It sounds like you don’t have much pain right now. In addition to opioid pain medications, some people find meditation to be helpful in reducing pain, or reducing the stress that pain causes. There are many free guided meditations available online that you could try. One meditation strategy is to close your eyes and think about a peaceful setting. Visualize what you are smelling, seeing, hearing, and feeling in that setting.  **ASB_6BM_G4_20**  It sounds like your pain has been fairly under control. Integrating exercise when you have a low level of pain can be a helpful strategy to pain management, in addition to your opioid pain medication. One way to incorporate exercise into your activities is to park further away from the entrance at the grocery store or using the stairs instead of the elevator.  **ASB_6BM_G4_21**  It sounds like your pain has been fairly low and under control. Some people find it helpful to use additional strategies to manage their pain. When people try to accomplish too much at one time, in the long run, it can cause their body to experience more pain than they would like. For example, try spacing out your errands instead of trying to do them all at once.  **ASB_6BM_G4_22**  It sounds like you have been experiencing a lower level of pain within the last day while taking your opioid pain medications. Ice tends to work better for new injuries by reducing inflammation, muscle spasms, and pain. However, if you find relief with ice for your older injuries, continue with what you have found to be successful.  **ASB_6BM_G4_23**  It sounds like your pain has been under control. People find it helpful to use additional strategies to manage their pain. Some people find that changes in the weather effect how much pain they experience. Try taking note of weather patterns and how they may overlap with your more painful days so that you may be better prepared to manage your pain.  **ASB_6BM_G4_24**  It sounds like you have been experiencing a lower level of pain within the last day while taking your opioid pain medication. In addition to opioid pain medications, some people use other strategies to manage pain. Staying flexible by stretching can keep you mobile longer and reduce some of the pain you are experiencing. One stretch that a lot of other people experiencing pain do is a side stretch. While standing or sitting, lift your right arm straight above your head. Without twisting, tilt your body slowly toward your left side as far as you can go and hold it for 5 seconds. Then bring your body back to center. Lower your right arm and try it with your left arm. Reach as far to your right side as you can and hold for 5 seconds. Repeat this three times to stretch out your back and get yourself moving each day.  **ASB_6BM_G4_25**  It seems you have had success in managing your pain in the last day while taking your opioid pain medications. In addition to opioid pain medications, some people find meditation to be helpful in reducing pain, or reducing the stress that pain causes. When you are feeling stressed out, try refocusing by making a list of things you can and cannot control. Focus on what you can control to make change, and accept what you cannot.  **ASB_6BM_G4_26**  It sounds like your pain has been under control and you’ve taken some medications as part of your pain management. Some people find activity pacing to be a helpful strategy in managing pain, in addition to their pain medication. This means to be careful in planning how much you try to do so that you don’t push yourself too hard. When doing activities like cleaning your house, try to break up the activity into manageable time. You may find it helpful to only clean one room every couple of hours so that you don’t get as tired after the activity.  **ASB_6BM_G4_27**  It sounds like your pain has been fairly low and under control. People who experience pain notice that it takes a toll on their mood. Some people find it helpful to write down three things that they are grateful for each day to maintain a positive outlook on life, and change the way they experience pain. This can also help to reduce the stress that pain can cause you.  **ASB_6BM_G4_28**  It sounds like you don’t have much pain right now. Having communication with others who also experience pain can be helpful, in addition to pain medication. Try joining an online group or forum for pain for comradery and pain management tips.  **ASB_6BM_G4_29**  It sounds like your pain has been fairly under control. When pain medication isn’t enough, heat is recommended for reoccurring injuries to increase your range of motion and improve the flexibility of tendons and ligaments. However, if you find that a combination of ice and heat works better for your pain, continue with what you have found to work. It’s best not to use ice or heat for more than 20 minutes at a time.  **ASB_6BM_G4_30**  It sounds like you don’t have much pain right now. Relaxation can be a great tool to help manage pain. Try taking an extended bath or shower. You may find it helpful to use Epsom Salt, pleasant aromas or calming music to help you relax even more. This can help with your stress related pain level as well as relieve some of your pain.  [After a message is played GO TO Section: Conclusion] |
| **Brief Message Group 5: MODERATE PAIN (3 - 6), OPIOID USE**  **ASB_6BM_G5_1**  It sounds like you have a moderate amount of pain right now, even with using opioid pain medication. Some people find that it can be helpful to track where the pain is, how bad it is, and what you have been doing to help manage the pain. That information could be helpful when discussing your pain with your doctor.  **ASB_6BM_G5_2**  It sounds like your pain has been bothering you, even with using opioid pain medications. Using ice and heat are common ways to manage muscle and joint-pain. Ice can help with inflammation and swelling. Heat can help increase blood flow to an area to promote healing. Ice or heat should not be applied for more than 20 minutes at a time.  **ASB_6BM_G5_3**  It sounds like you have a moderate amount of pain right now, even with using opioid pain medications. Some people find activity pacing to be a helpful strategy in managing pain. This means to be careful in planning how much you try to do so that you don’t push yourself too hard. For example, rather than trying to do all of a physical task, like washing the dishes all at once, try breaking it up into shorter periods.  **ASB_6BM_G5_4**  It sounds like your pain has been bothering you, even with using opioid pain medications. Increasing your strength through exercise can help reduce pain over time. Aim to have at least 20 minutes of exercise a day, if you don’t already meet this goal. This could be split into a couple of times over the day, like a five minute walk in the morning, a ten minute walk after lunch, and another five minute walk in the evening.  **ASB_6BM_G5_5**  It sounds like you have a moderate amount of pain right now, even with using opioid pain medications. Some people find meditation helpful in reducing pain, or reducing the stress that pain causes. One way to do this is called breathing from your belly. To do this, you place one hand on your chest and your other hand on your stomach while sitting or lying down. You then breathe deeply into your stomach and you will notice the hand on your stomach rising as the one on your chest falls.  **ASB_6BM_G5_6**  It sounds like your pain has been bothering you, even with using opioid pain medications. Some people find that distractions, such as reading, watching tv, listening to music, or other activities, can be helpful for taking attention away from pain.  **ASB_6BM_G5_7**  It sounds like you have a moderate amount of pain right now, even with using opioid pain medications. Sometimes people find doing meditation or relaxation techniques to be helpful in reducing pain, or reducing the stress that pain causes. As an exercise, try squeezing your right hand into a fist and holding it for five seconds and then releasing. Notice how the sensation changes. Next try it with your left hand. In doing this you notice that the body can hold tension and release it. You can do this progressive muscle relaxation with all muscle groups of your body.  **ASB_6BM_G5_8**  It sounds like your pain has been bothering you, even with using opioid pain medications. Asking for support from others is another way to have your needs met so your pain does not get worse.  **ASB_6BM_G5_9**  It sounds like you have a moderate amount of pain right now, even with using opioid pain medications. Some people find meditation to be helpful in reducing pain, or reducing the stress that pain causes. There are many free guided meditations available online that you could try. One meditation strategy is to close your eyes and think of relaxing each part of your body, one at a time.  **ASB_6BM_G5_10**  It sounds like your pain has been bothering you, even with using opioid pain medication. Contacting your doctor’s office may be helpful to get additional tips for managing moderate pain like yours. If you do not have a regular doctor, you could consider talking to friends or relatives that also experience pain, or looking up pain management options online.  **ASB_6BM_G5_11**  It sounds like you have a moderate amount of pain right now, even with using opioid pain medications. People who experience moderate levels of pain notice that it takes a toll on their mood. Some people find it helpful to do little acts of kindness for other people each day and write them down. This can help to maintain a positive outlook on life and reduce the stress that pain can cause you.  **ASB_6BM_G5_12**  It sounds like your pain has been bothering you, even with using opioid pain medications. When people try to accomplish too much at one time, in the long run, it can cause their body to experience more pain than they would like. Try setting a timer for 15 minutes when doing household chores or yard work in order to take breaks throughout your day.  **ASB_6BM_G5_13**  It sounds like you have a moderate amount of pain right now, even with using opioid pain medications. Exercise can help someone experiencing pain by loosening up stiff joints. Utilizing some of the items you have at home, like soup cans or juice containers, could be helpful substitutes for strength training weights. Try doing ten bicep curls with soup cans each day to get your muscles moving and build your strength.  **ASB_6BM_G5_14**  It sounds like your pain has been bothering you, even with using opioid pain medications. In addition to opioid pain medications, some people use other strategies to manage pain. Some people find breathing exercises to be helpful in reducing pain, or reducing stress and anxiety related to pain. Let’s try one of these, Start by breathing in through your nose to a count of 5. 1-2-3-4-5-Now hold the breath for a count of 5. 1-2-3-4-5 Lastly, slowly exhale through your mouth for a count of 5. 1-2-3-4-5 Repeat this a few times. This activity can be done anywhere and it is a quick way to find some relief from anxiety and stress.  **ASB_6BM_G5_15**  It sounds like you have a moderate amount of pain right now, even with using opioid pain medications. Today find a time to do one of your favorite activities when you notice signs that pain is increasing. This may help you refocus on an activity that you enjoy, and get your mind off of the pain.  **ASB_6BM_G5_16**  It sounds like your pain has been bothering you, even with using opioid pain medication. Sometimes people find doing meditation or relaxation techniques to be helpful in reducing pain, or reducing the stress that pain causes. As an exercise, try squeezing your shoulders by raising them up towards your ears. Hold it for 5 seconds and then release it. Notice how relaxed your shoulders feel now. You may want to try this throughout the day, as we can hold a lot of tension in our shoulders without noticing it.  **ASB_6BM_G5_17**  It sounds like you have a moderate amount of pain right now, even with using opioid pain medications. Try asking someone to be your work out buddy. Exercise can help someone experiencing pain by loosening up stiff joints, and having somebody to work out with can keep you motivated and make exercise more enjoyable.  **ASB_6BM_G5_18**  It sounds like your pain has been bothering you, even with using opioid pain medications. Getting enough sleep is important to managing pain. When your pain is bad, it might be from not getting enough sleep the night before. It could be helpful to track your sleep and look at how it affects your pain levels the next day.  **ASB_6BM_G5_19**  It sounds like you have a moderate amount of pain right now, even with using opioid pain medications. Some people find meditation to be helpful in reducing pain, or reducing the stress that pain causes. There are many free guided meditations available online that you could try. One meditation strategy is to close your eyes and think about a peaceful setting. Visualize what you would be smelling, seeing, hearing, and feeling in that setting.  **ASB_6BM_G5_20**  It sounds like your pain has been bothering you, even with using opioid pain medications. Making time for exercise can be challenging when you are experiencing pain. One way to incorporate exercise into your activities is to park further away from the entrance at the grocery store or using the stairs instead of the elevator.  **ASB_6BM_G5_21**  It sounds like you have a moderate amount of pain right now, even with using opioid pain medication. When people try to accomplish too much at one time, in the long run, it can cause their body to experience more pain than they would like. For example, try spacing out your errands instead of trying to do them all at once.  **ASB_6BM_G5_22**  It sounds like your pain has been bothering you, even with using opioid pain medication. Ice tends to work better for new injuries by reducing inflammation, muscle spasms, and pain. However, if you find relief with ice for your older injuries, continue with what you have found to be successful.  **ASB_6BM_G5_23**  It sounds like you have a moderate amount of pain right now, even with using opioid pain medication. Some people find that changes in the weather effect how much pain they experience. Try taking note of weather patterns and how they may overlap with your more painful days so that you may be better prepared to manage your pain.  **ASB_6BM_G5_24**  It sounds like your pain has been bothering you, even with using opioid pain medications. Staying flexible by stretching can keep you mobile longer and reduce some of the pain you are experiencing. One stretch that a lot of other people experiencing pain do is a side stretch. While standing or sitting, lift your right arm straight above your head. Without twisting, tilt your body slowly toward your left side as far as you can go and hold it for 5 seconds. Then bring your body back to center. Lower your right arm and try it with your left arm. Reach as far to your right side as you can and hold for 5 seconds. Repeat this three times to stretch out your back and get yourself moving each day.  **ASB_6BM_G5_25**  It sounds like you have a moderate amount of pain right now, even with using opioid pain medications. In addition to opioid pain medications, some people find meditation to be helpful in reducing pain, or reducing the stress that pain causes. When you are feeling stressed out, try refocusing by making a list of things you can and cannot control. Focus on what you can control to make change, and accept what you cannot.  **ASB_6BM_G5_26**  It sounds like your pain has been bothering you, even with using opioid pain medication. Some people find activity pacing to be a helpful strategy in managing pain. This means to be careful in planning how much you try to do so that you don’t push yourself too hard. When doing activities like cleaning your house, try to break up the activity into manageable time. You may find it helpful to only clean one room every couple of hours so that you don’t get as tired after the activity.  **ASB_6BM_G5_27**  It sounds like you have a moderate amount of pain right now, even with using opioid pain medications. People who experience moderate levels of pain notice that it takes a toll on their mood. Some people find it helpful to write down three things that they are grateful for each day to maintain a positive outlook on life, and change the way they experience pain. This can also help to reduce the stress that pain can cause you.  **ASB_6BM_G5_28**  It sounds like your pain has been bothering you, even with using opioid pain medication. Having communication with others who also experience pain can be helpful. Try joining an online group or forum for pain for comradery and pain management tips.  **ASB_6BM_G5_29**  It sounds like you have a moderate amount of pain right now, even with using opioid pain medications. Heat is recommended for reoccurring injuries to increase your range of motion and improve the flexibility of tendons and ligaments. However, if you find that a combination of ice and heat works better for your pain, continue with what you have found to work. It’s best not to use ice or heat for more than 20 minutes at a time.  **ASB_6BM_G5_30**  It sounds like your pain has been bothering you, even with using opioid pain medications. Relaxation can be a great tool to help manage pain. Try taking an extended bath or shower. You may find it helpful to use Epsom Salt, pleasant aromas or calming music to help you relax even more. This can help your pain related stress level as well as relieve some of your pain.  [After a message is played GO TO Section: Conclusion] |
| **Brief Message Group 6: HIGH PAIN (> 6), OPIOID USE**  **ASB_6BM_G6_1**  It sounds like you have been in a lot of pain over the last day, even with using opioid pain medications. Some people find that it to be helpful to track where the pain is, how bad it is, and what you have been doing to manage the pain. That information could be helpful when discussing your pain with your doctor.  **ASB_6BM_G6_2**  It sounds like your pain has been bothering you a lot over the last day, even with using opioid pain medications. Using ice and heat are common ways to manage muscle and joint-pain. Ice can help with inflammation and swelling. Heat can help increase blood flow to an area to promote healing. Ice or heat should not be applied for more than 20 minutes at a time.  **ASB_6BM_G6_3**  It sounds like you have been in a lot of pain over the last day, even with using opioid pain medications. Some people find activity pacing to be a helpful strategy in managing pain. This means to be careful in planning how much you try to do so that you don’t push yourself too hard.For example, rather than trying to do all of a physical task, like washing the dishes all at once, try breaking it up into shorter periods.  **ASB_6BM_G6_4**  It sounds like your pain has been bothering you a lot over the last day, even with using opioid pain medications. Increasing your strength through exercise can help reduce pain over time. Aim to have at least 20 minutes of exercise a day, if you don’t already meet this goal. This could be split into a couple times over the day, like a five minute walk in the morning, a ten minute walk after lunch, and another five minute walk in the evening.  **ASB_6BM_G6_5**  It sounds like you have been in a lot of pain over the last day, even with using opioid pain medications. Some people find meditation helpful in reducing pain, or reducing the stress that pain causes, even if only a little. One way to do this is called breathing from your belly. To do this, you place one hand on your chest and your other hand on your stomach while sitting or lying down. You then breathe deeply into your stomach and you will notice that the hand on your stomach rising as the one on your chest falls.  **ASB_6BM_G6_6**  It sounds like your pain has been bothering you a lot over the last day, even with using opioid pain medications. Some people find that distractions, such as reading, watching tv, listening to music, or other activities, can be helpful for taking attention away from pain.  **ASB_6BM_G6_7**  It sounds like you have been in a lot of pain over the last day, even with using opioid pain medications. Sometimes people find doing meditation or relaxation techniques to be helpful in reducing pain, or reducing the stress that pain causes, even if only a little. As an exercise, try squeezing your right hand into a fist and holding it for five seconds. Then releasing it. Notice how the sensation changes. Next try it with your left hand. In doing this you notice that the body can hold tension and release it. You can do this progressive muscle relaxation with all the parts of your body.  **ASB_6BM_G6_8**  It sounds like your pain has been bothering you a lot over the last day, even with using opioid pain medications. Asking for support from others is another way to have your needs met so your pain does not get worse.  **ASB_6BM_G6_9**  It sounds like you have been in a lot of pain over the last day, even with using opioid pain medications. Some people find meditation to be helpful in reducing pain, or reducing the stress that pain causes, even if only a little. There are many free guided meditations available online that you could try. One meditation strategy is to close your eyes and think of relaxing each part of your body, one at a time.  **ASB_6BM_G6_10**  It sounds like your pain has been bothering you a lot over the last day, even with using opioid pain medications. When pain becomes very high, it may be hard to manage alone. Contacting your doctor’s office may help provide you with options for managing strong pain like yours. If you do not have a regular doctor, you could consider talking to friends or relatives that also experience pain, or looking up pain management options online.  **ASB_6BM_G6_11**  It sounds like you have been in a lot of pain over the last day, even with using opioid pain medications. People who experience high levels of pain notice that it takes a toll on their mood. Some people find it helpful to do little acts of kindness for other people each day and write them down. This can also help to maintain a positive outlook on life and reduce the stress that pain can cause you.  **ASB_6BM_G6_12**  It sounds like your pain has been bothering you a lot over the last day, even with using opioid pain medication. When people try to accomplish too much at one time, in the long run, it can cause their body to experience more pain than they would like. Try setting a timer for 15 minutes when doing household chores or yard work in order to take breaks throughout your day.  **ASB_6BM_G6_13**  It sounds like you have been in a lot of pain over the last day, even with using opioid pain medication. Exercise can help someone experiencing pain by loosening up stiff joints. Utilizing some of the items you have at home, like soup cans or juice containers, could be helpful substitutes for strength training weights. Try doing ten bicep curls with soup cans each day to get your muscles moving and build your strength.  **ASB_6BM_G6_14**  It sounds like your pain has been bothering you a lot over the last day, even with using opioid pain medications. In addition to opioid pain medications, some people use other strategies to manage pain. Some people find breathing exercises to be helpful in reducing pain, or reducing stress and anxiety related to pain. Let’s try one of these, Start by breathing in through your nose to a count of 5. 1-2-3-4-5 Now hold the breath for a count of 5. 1-2-3-4-5 Lastly, slowly exhale through your mouth for a count of 5. 1-2-3-4-5 Repeat this a few times. This activity can be done anywhere and it is a quick way to find some relief from anxiety and stress.  **ASB_6BM_G6_15**  It sounds like you have been in a lot of pain over the last day, even with using opioid pain medication. Today find a time to do one of your favorite activities when you notice signs that pain is increasing, or when you want a distraction. This may help you refocus on an activity that you enjoy, and keep your mind off of the pain.  **ASB_6BM_G6_16**  It sounds like your pain has been bothering you a lot over the last day, even with using opioid pain medications. Sometimes people find doing meditation or relaxation techniques to be helpful in reducing pain some, or reducing the stress that pain causes. As an exercise, try squeezing your shoulders by raising them up towards your ears. hold it for 5 seconds and then release it. Notice how relaxed your shoulders feel now. You may want to try this throughout the day, as we can hold a lot of tension in our shoulders without noticing it.  **ASB_6BM_G6_17**  It sounds like you have been in a lot of pain over the last day, even with using opioid pain medications. Try asking someone to be your work out buddy. Exercise can help someone experiencing pain by loosening up stiff joints, and having somebody to work out with can keep you motivated and make exercise more enjoyable.  **ASB_6BM_G6_18**  It sounds like your pain has been bothering you a lot over the last day, even with using opioid pain medications. Getting enough sleep is important for managing pain. When your pain is bad, it might be from not getting enough sleep the night before. It could be helpful to track your sleep and look at how it affects your pain levels the next day.  **ASB_6BM_G6_19**  It sounds like you have been in a lot of pain over the last day, even with using opioid pain medications. Some people find meditation to be helpful in reducing pain, or reducing the stress that pain causes. There are many free guided meditations available online that you could try. One meditation strategy is to close your eyes and think about a peaceful setting. Visualize what you are smelling, seeing, hearing, and feeling in that setting.  **ASB_6BM_G6_20**  It sounds like your pain has been bothering you a lot over the last day, even with using opioid pain medications. Making time for exercise can be challenging when you are experiencing pain. One way to incorporate exercise into your activities is to park further away from the entrance at the grocery store or using the stairs instead of the elevator.  **ASB_6BM_G6_21**  It sounds like you have been experiencing some significant pain in the last day while taking opioid pain medications. When people try to accomplish too much at one time, in the long run, it can cause their body to experience more pain than they would like. For example, try spacing out your errands instead of trying to do them all at once.  **ASB_6BM_G6_22**  It sounds like your pain has been bothering you a lot over the last day, even with using opioid pain medications. Ice tends to work better for new injuries by reducing inflammation, muscle spasms, and pain. However, if you find relief with ice for your older injuries, continue with what you have found to be successful.  **ASB_6BM_G6_23**  It sounds like you have been in a lot of pain over the last day, even with using opioid pain medications. Some people find that changes in the weather effect how much pain they experience. Try taking note of weather patterns and how they may overlap with your more painful days so that you may be better prepared to manage your pain.  **ASB_6BM_G6_24**  It sounds like your pain has been bothering you a lot over the last day, even with using opioid pain medications. Staying flexible by stretching can keep you mobile longer and reduce some of the pain you are experiencing. One stretch that a lot of other people experiencing pain do is a side stretch. While standing or sitting, lift your right arm straight above your head. Without twisting, tilt your body slowly toward your left side as far as you can go and hold it for 5 seconds. Then bring your body back to center. Lower your right arm and try it with your left arm. Reach as far to your right side as you can and hold for 5 seconds. Repeat this three times to stretch out your back and get yourself moving each day.  **ASB_6BM_G6_25**  It sounds like you have been in a lot of pain over the last day, even with using opioid pain medications. In addition to opioid pain medications, some people find meditation to be helpful in reducing pain some, or reducing the stress that pain causes. When you are feeling stressed out, try refocusing by making a list of things you can and cannot control. Focus on what you can control to make change, and accept what you cannot.  **ASB_6BM_G6_26**  It sounds like your pain has been bothering you a lot over the last day, even with using opioid pain medications. Some people find activity pacing to be a helpful strategy in managing pain. This means to be careful in planning how much you try to do so that you don’t push yourself too hard. When doing activities like cleaning your house, try to break up the activity into manageable time. You may find it helpful to only clean one room every couple of hours so that you don’t get as tired after the activity.  **ASB_6BM_G6_27**  It sounds like you have been in a lot of pain over the last day, even with using opioid pain medications. People who experience high levels of pain notice that it takes a toll on their mood. Some people find it helpful to write down three things that they are grateful for each day to maintain a positive outlook on life, and change the way they experience pain. This can also help to reduce the stress that pain can cause you.  **ASB_6BM_G6_28**  It sounds like your pain has been bothering you a lot over the last day, even with using opioid pain medications. Having communication with others who also experience pain can be helpful. Try joining an online group or forum for pain for comradery and pain management tips.  **ASB_6BM_G6_29**  It sounds like you have been in a lot of pain over the last day, even with using opioid pain medications. Heat is recommended for reoccurring injuries to increase your range of motion and improve the flexibility of tendons and ligaments. However, if you find that a combination of ice and heat works better for your pain, continue with what you have found to work. It’s best not to use ice or heat for more than 20 minutes at a time.  **ASB_6BM_G6_30**  It sounds like your pain has been bothering you a lot over the last day, even with using opioid pain medications. Relaxation can be a great tool to help manage pain. Try taking an extended bath or shower. You may find it helpful to use Epsom Salt, pleasant aromas, or calming music to help you relax even more. This can help your stress related pain level as well as relieve some of your pain.  [After a message is played GO TO Section: Conclusion] |

# Extended Message

| **ASB_7EM0**  [Play an extended question from set 1, an extended message from set 2, and then the extended summary in set 3, before going to the Section: Conclusion] |
| --- |
| **Extended Content: messages are based on motivational enhancement therapist manual** |

| **SET 1: Extended Question** |
| --- |
| **ASB_7EM_1Q_W00_Tool0**  *** NOTE: Participants may receive the Tools for Change question set up to 3 times throughout enrollment ***  Next, I would like to talk through how you manage your pain, and health overall. We have come up with three general categories of the types of things that people have shared with us that helps them start, or continue to, manage their pain symptoms. Please listen to the following list and the associated number. After you have heard all three options, please press the number corresponding to the category would be most helpful to you with managing your pain or opioid use.  1 – Speak with a physician or therapist.  2 – Develop coping skills or alternative options for my pain.  3 – Work to improve my health.  [IF 1, GO TO ASB_7EM_1Q_W01_Tool1]  [IF 2, GO TO ASB_7EM_1Q_W02_Tool2]  [IF 3, GO TO ASB_7EM_1Q_W03_Tool3] |
| **ASB_7EM_1Q_W01_Tool1**  You have chosen Speak with a physician or therapist as the most helpful idea at this time. There are a lot of different topics, concerns, and questions that people have around their pain or opioid use. Which of the following options relate to your desire to speak with a physician or therapist? Please press the number that corresponds best to what you think.  1 – Talk to my doctor about my pain or medication use.  2 – Discuss the side effects I experience with my medications.  3 – Recognize withdrawal symptoms when reducing or stopping opioid medications.  4 – Discuss ways to manage pain that I am not already using.  5 – Other topics.  [GO TO ASB_7EM_1Q_W01_Tool1_Msg] |
| **ASB_7EM_1Q_W01_Tool1_Msg**  Being able to have an open and positive relationship with a physician or therapist is important to managing your pain. Consider making an appointment to discuss your questions.Some people find it helpful to write down their questions as they think of them at home, so they can remember everything they want to go over with their provider at the appointment.  [GO TO SET 2: Extended Message] |
| **ASB_7EM_1Q_W02_Tool2**  You have chosen Develop coping skills or alternative options for my pain as the most helpful idea at this time. Here is a list of options that other people have used to help manage their pain or other problems. Please press the number that corresponds to something that you’d be interested in trying, or have used in the past and could use again.  1 – Exercise, stretching or yoga.  2 – Spend time with people who I find encouraging.  3 – Take medication according to the instructions from my doctor.  4 – Read, write in a journal, or watch television.  5 – Spend some time doing a hobby I enjoy, or trying a new one.  6 – Something else  Please press a number at this time.  [GO TO ASB_7EM_1Q_W01_Tool2_Msg] |
| **ASB_7EM_1Q_W02_Tool2_Msg**  People have some tricks and tools that help them get through their pain. It can be helpful to think about other options that weren’t listed that might be helpful when coping with your pain. Consider trying something new to manage your pain today.  [GO TO SET 2: Extended Message] |
| **ASB_7EM_1Q_W03_Tool3**  You have chosen Work to improve my health as the most useful idea at this time. There are many options that people can do to improve their health. For many people experiencing pain, what you do today influences how you feel tomorrow. Making one small change today can mean big changes over time. Press a number that corresponds to a change you would like to make.  1 – Start an exercise, stretching or yoga routine.  2 – Reduce my stress level by meditating or deep breathing.  3 – Change one health habit, like how much I drink or smoke.  4 – Concentrate on eating healthier foods, less salt, or less fast food.  5 – Drink less soda or eat less sugary foods.  6 – Do things that help me connect with myself or my spiritual side.  7 – Find enjoyable hobbies.  8 – Something else.  Please press a number at this time.  [GO TO ASB_7EM_1Q_W01_Tool3_Msg] |
| **ASB_7EM_1Q_W03_Tool3_Msg**  Consider one smaller goal you could accomplish today that could get you one step closer to the goal you want to achieve. Change doesn’t have to happen all at once. It should be a gradual process.  [GO TO SET 2: Extended Message] |
| **ASB_7EM_1Q_W04-7_Behav_Intro**  **ASB_7EM_1Q_W04_Behav1**  Next, we would like to assess how you are feeling about your pain. How confident do you feel about your ability to manage your current pain? Please rank your response on a scale of 0 to 10, where 0 means not at all confident, and 10 means very confident. If you do not have a problem with pain right now, select 10.  [GO TO SET 2: Extended Message] |
| **ASB_7EM_1Q_W05_Behav2**  Next, we would like to assess your future outlook for your pain. How important is it to you today to find ways to improve how you manage your pain in the future? Please rate your response on a scale of 0 to 10, where 0 means not at all important, and 10 means very important.  [GO TO SET 2: Extended Message] |
| **ASB_7EM_1Q_W06_Behav3**  Next, we would like to assess your pain care. Today, how confident are you that you can use your opioids safely? Please rate your response on a scale of 0 to 10, where 0 means not at all confident, and 10 means very confident.  [GO TO SET 2: Extended Message] |
| **ASB_7EM_1Q_W07_Behav4**  Next, we would like to assess your thoughts on use of opioids. How likely is it that your use of opioids to manage pain will be different in one month from now than it is today? Please rank your response on a scale of 0 to 10, where 0 means not at all likely, and 10 means very likely.  [GO TO SET 2: Extended Message] |
| **ASB_7EM_1Q_W08-12_Info_Intro**  **ASB_7EM_1Q_W08-12_Info_End**  **ASB_7EM_1Q_W08_Info1**  Next, we would like to share some information and find out how helpful you find it to be. This will help us to know what kinds of information will be helpful to other people in the future.  Overdoses due to opioids have been brought up a lot lately in the news. An overdose is when the body has had too much of a substance or subtances. Reactions can range from passing out or throwing up, to fatality. This has a lot of people worried about their own risk. There are things that people can do to lower their risk of overdose when they use opioids, such as avoiding using opioids the same day as other substances, like alcohol and sedative medications, and not doing anything to change the pills themselves like crushing them.  Please rank how helpful this information is to you on a scale of 0 to 10, where 0 is not helpful at all, and 10 as very helpful.  [GO TO SET 2: Extended Message] |
| **ASB_7EM_1Q_W09_Info2**  Next, we would like to share some information and find out how helpful you find it to be. This will help us to know what kinds of information might be helpful to other people in the future.  People develop what is called tolerance when they use opioids consistently over time, which means that the person’s body gets used to a certain dose, and the person needs to take a higher dose to get the same pain relief. If you’re finding that you may be developing tolerance, talk to your doctor or nurse. Having breaks in opioid use can help to avoid developing tolerance, but it is also important to go back to taking a smaller dose after a break if a person developed tolerance before because the person’s body is no longer used to the previous dose and at a greater risk of overdose.  Please rank how helpful this information is to you on a scale of 0 to 10, where 0 is not helpful at all, and 10 as very helpful.  [GO TO SET 2: Extended Message] |
| **ASB_7EM_1Q_W10_Info3**  Next, we would like to share some information and find out how helpful you find it to be. This will help us to know what kinds of information might be helpful to other people in the future.  Naloxone, also called Narcan, is a medication that can reverse the effects of an opioid overdose. It is what is used in the emergency room to treat opioid overdoses, and some first responders, like paramedics and police, also carry it. There are new programs to make naloxone available to members of the community who are interested in having it in order to be able to help if they witness someone else having an overdose. Common signs of overdosing on opioid medications are extreme sleepiness or nodding off, breathing problems, and small pupils, among others.  Please rank how helpful this information is to you on a scale of 0 to 10, where 0 is not helpful at all, and 10 as very helpful.  [GO TO SET 2: Extended Message] |
| **ASB_7EM_1Q_W11_Info4**  Next, we would like to share some information and find out how helpful you find it to be. This will help us to know what kinds of information might be helpful to others in the future.  Studies have shown that certain types of counseling, such as a form called cognitive behavioral therapy, can reduce pain. In this type of treatment, the treatment provider helps the patient learn tools and strategies for how they go about their day in order to reduce pain, and ways to understand pain. It can be done with or without other pain management strategies.  Please rate how helpful this information is to you on a scale of 0 to 10, where 0 is not helpful at all, and 10 as very helpful.  [GO TO SET 2: Extended Message] |
| **ASB_7EM_1Q_W12_Info5**  Next, we would like to share some information and find out how helpful you find it to be. This will help us to know what kinds of information might be helpful to other people in the future.  If you take opioids for a while and then stop, you can experience withdrawal. Symptoms of opioid withdrawal include stomach cramps, nausea, vomiting, chills, and diarrhea. The intensity of these symptoms can be reduced by slowly reducing the dose you are taking of the opioid, rather than stopping all at once.  Please rank how helpful this information is to you on a scale of 0 to 10, where 0 is not helpful at all, and 10 as very helpful.  [GO TO SET 2: Extended Message] |

| **SET 2: Extended Message** |
| --- |
| **ASB_7EM_2M_G0**  [Play an extended message from below based on reported pain level (ASB_2Q1), opioid use (ASB_2Q2), medical misuse (ASB_3Q1 & ASB_3Q2), and non-medical misuse (ASB_3Q3).]   \|  \| ASB_2Q1 \| ASB_2Q2 \| ASB_3Q1 \| ASB_3Q2 \| ASB_3Q3 \| \| --- \| --- \| --- \| --- \| --- \| --- \| \| No OA Use: G1 \| 0,1,2 \| 0 \| - \| - \| - \| \| No OA Use: G2 \| 3,4,5,6 \| 0 \| - \| - \| - \| \| No OA Use: G3 \| 7,8,9,10 \| 0 \| - \| - \| - \| \| Medical OA Use: G4 \| <3 \| ≥1 \| 3 \| 3 \| 3 \| \| Medical OA Misuse: G5 \| <3 \| ≥1 \| 1,2 \| 1,2,3 \| 3 \| \| Medical OA Misuse: G5 \| <3 \| ≥1 \| 1,2,3 \| 1,2 \| 3 \| \| Non-Medical OA Misuse: G6 \| <3 \| ≥1 \| 1,2,3 \| 1,2,3 \| 1,2 \| \| Medical OA Use: G7 \| ≥3 \| ≥1 \| 3 \| 3 \| 3 \| \| Medical OA Misuse: G8 \| ≥3 \| ≥1 \| 1,2 \| 1,2,3 \| 3 \| \| Medical OA Misuse: G8 \| ≥3 \| ≥1 \| 1,2,3 \| 1,2 \| 3 \| \| Non-Medical OA Misuse: G9 \| ≥3 \| ≥1 \| 1,2,3 \| 1,2,3 \| 1,2 \| |
| **Group 1: LOW PAIN (< 3), NO OPIOID USE**  **ASB_7EM_2M_G1_01**  It sounds like you have had a low level of pain in the last day and did not take any opioid pain medications. Some people find it helpful to use mindful breathing in managing pain and reducing stress. In case you are interested in trying it, here is what it is like. Place one hand on your chest and one on your belly. Try to focus on breathing deeply into your stomach while avoiding any movement in your shoulders. Concentrate on your breathing. Feel your stomach rise and fall.  **ASB_7EM_2M_G1_02**  It sounds like you have been experiencing a lower level of pain within the last day and did not take any opioid pain medication. Having someone to talk to about your pain or stressful situations can be helpful. Asking for support from others is a way to have your physical and emotional needs met. Think of someone in your life that has been supportive and consider reaching out to them to talk about your pain today.  **ASB_7EM_2M_G1_03**  It sounds like you haven’t needed to take any opioid pain medications in the last day. Regular exercise can help keep your pain lower over time, in addition to all the other benefits to your health. Try taking a short walk around the block or inside the house. Building up to exercising 20 minutes a day has been shown to have many benefits including reducing pain. If you are already meet this goal regularly, adding in more stretching or strength training, or another goal you might want for yourself, can be helpful. Even though there may be a little discomfort and muscle pain for a short period of time when you are first getting into a new exercise routine, it will be worth it for avoiding future pain.  **ASB_7EM_2M_G1_04**  It sounds like you have been experiencing a lower level of pain within the last day. Here’s a tool you may wish to use the next time you have a lot of stress or pain. Some people find relaxation helpful in managing pain. One method is to close your eyes and concentrate on relaxing your whole body by squeezing and relaxing one body part at a time. Try squeezing your right fist as hard as you can and relaxing it after five seconds. Then proceed to your left fist. Then your feet, one at a time, moving from one body part to the next on your way to your head. Once you have done this to your entire body, try being still and feeling the sense of relaxation for a few minutes.  **ASB_7EM_2M_G1_05**  It sounds like you have been successful in managing your pain in the last day and have not taken any opioid pain medication. A big part of pain and stress management is having high sleep quality. Think about what it takes for you to have a good night’s sleep. What does your routine look like before bed? How can you reinforce this routine to ensure you have more good night’s sleep? What could you include to improve your routine? Thinking about what you need for good sleep quality can help reduce your pain and make sleeping more enjoyable.  **ASB_7EM_2M_G1_06**  It sounds like you have been able to reduce the pain you had in the past and did not need to take opioid pain medications. Some people have found that purposefully celebrating or enjoying the positive things in their lives to be helpful. What are some things that are going well for you right now? Take some time for yourself to celebrate your life, work, family, friends or loved ones. Stop and enjoy a good meal, a fun activity, or a kind “hello”.  **ASB_7EM_2M_G1_07**  It sounds like you don’t have much pain right now and you didn’t take any opioid pain medication in the last day. One strategy that other people have found helpful to avoid worsening a chronic pain problem or recovering from acute pain includes pacing how much they do at one time so that they don’t push themselves too hard. It is sometimes easier on your body to take several small breaks during your daily activities instead of pushing through only to rest at the end. You may find your body is quicker to recover and you will experience less pain if you take short breaks regularly.  **ASB_7EM_2M_G1_08**  Managing your pain or other stresses day to day is not always an easy task. You told us that your pain was low today, and you must have been persistent at finding something that helps you be successful in getting past the pain from the problem that brought you to the emergency room. That’s great, and you should be very proud. It might be useful to take a moment to reflect on what you’ve done well.  **ASB_7EM_2M_G1_09**  It sounds like you don’t have much pain right now and you didn’t take any opioid pain medication in the last day. Some people find it helpful to write about their daily thoughts and activities in a journal. By writing things down, we can often find ourselves more at peace with our difficulties, feel more grounded, or see patterns we hadn’t noticed. If you don’t already have one, perhaps you could start a journal today. What kinds of things might be helpful to write down?  **ASB_7EM_2M_G1_10**  It sounds like you have found some ways to keep your pain low. Reviewing how you accomplished this in the time since you visited the emergency room could be useful in continuing your success. Take note of strategies you may be using to avoid pain, and think about ways you already incorporate these strategies into your life consistently.  **ASB_7EM_2M_G1_11**  You seem to be doing a great job of managing your pain over the last day, and haven’t had to take any opioid pain medications. Sometimes our personal self-image can have a big impact on how we feel. A quick and proven way to improve our own feelings of self-worth is to perform an act of kindness. What is something nice you could do today for a friend, someone you love, or even a complete stranger? Afterwards, note how it makes you feel, not just emotionally, but physically.  **ASB_7EM_2M_G1_12**  It sounds like your pain has not bothered you in the last day. Pain can come and go. If you were to have pain again, one technique for pain management is the ice and heat methods. In general, use ice to treat acute injury or acute pain. On the other hand, use heat to treat muscle or joint stiffness and chronic pain, but not bruises, swellings, or open wounds.  [After an extended message is played GO TO SET 3: Extended Summary] |
| **Group 2: MODERATE PAIN (3 – 6), NO OPIOID USE**  **ASB_7EM_2M_G2_01**  It sounds like you have been experiencing some pain in the last day and did not take any opioid pain medications. Some people find it helpful to use mindful breathing in managing pain. Here is one strategy for mindful breathing. Try placing a hand on your chest and one on your belly. Try to focus on breathing deeply into your stomach and avoiding any movement in your shoulders. Feel your stomach rise and fall. This should reduce stress and clear your mind.  **ASB_7EM_2M_G2_02**  It sounds like you have been experiencing a moderate level of pain within the last day and did not take any opioid pain medications. Asking for support from others is another way to have your physical and emotional needs met, which can help you to keep your pain level down. Is there someone in your life that has been supportive to you in the past? Could you consider reaching out to them in the next few days? Having someone to talk about your emotions around your pain can help reduce your pain and your stress.  **ASB_7EM_2M_G2_03**  It sounds like you have found some ways to manage your pain in the last day and haven’t taken any opioid pain medication. A lot of people have a belief that exercise can only make pain worse. Even though there may be some muscle pain for a short period of time, regular exercise can help keep your pain lower over time. Try taking a walk around the block or inside the house. Building up to exercising 20 minutes a day has been shown to have many benefits including reducing pain. If you already meet this goal regularly, adding in more stretching, strength training, or another goal could be helpful.  **ASB_7EM_2M_G2_04**  It sounds like you have been dealing with some pain within the last day and didn’t take any opioid pain medications. Some people find relaxation helpful in managing pain. One method is to close your eyes and concentrate on relaxing your whole body by squeezing and relaxing one body part at a time. Try squeezing your right fist as hard as you can and relaxing it after five seconds. Then do this your left fist. Then your feet, one at a time, moving from one body part to the next on your way to your head. Once you have done this to your entire body, try being still and feel the sense of relaxation for a few minutes.  **ASB_7EM_2M_G2_05**  It seems you have had moderate amount of pain in the last day and have not taken any opioid pain medications. A big part of pain management is getting enough good sleep. Think about how well you slept last night. What did you find that wasn’t helpful to your sleep? What did you notice that might have contributed to making your night of sleep better? Consider developing a routine before bed to help go to sleep faster and deeper.  **ASB_7EM_2M_G2_06**  It sounds like you are experiencing some pain and haven’t taken any opioid pain medication. Overcoming the pain you are experiencing isn’t always easy to do, but finding multiple things you can do that reduce your pain can be helpful. When someone experience pain day in and day out, they can get a negative attitude about it, and understandably become irritable or get frustrated easily. Keeping your thoughts positive about your pain has been shown to help with facing pain each day. Try thinking of four or five positive statements about yourself or things you enjoy that you can repeat to stay motivated with overcoming your pain.  **ASB_7EM_2M_G2_07**  It sounds like you’ve been some pain but you didn’t take any opioid pain medications in the last day. It could be helpful to think about things you’ve been doing over the last couple of days that helped you avoid having more pain than you have now. Some people find it helpful to pace how much they do at one time so that they don’t push themselves too hard. It is sometimes easier on your body to take several small breaks during your daily activities instead of pushing through only to rest after. You may find your body is quicker to recover and you will experience less pain if you take several breaks throughout the course of the day.  **ASB_7EM_2M_G2_08**  Managing pain can be difficult, but you don’t have to go at it alone. Talking to a healthcare professional about your pain could be useful to learn about all of your options to reduce your pain.  **ASB_7EM_2M_G2_09**  It sounds like you have some pain, but that you’ve been managing it without opioid pain medications. Some people find it helpful to write about their daily thoughts and activities in a journal. By writing things down, we can often find ourselves more at peace with our difficulties, feel more grounded, or see patterns we hadn’t noticed about our pain. If you don’t already have one, perhaps you could start a journal today. What kinds of things might be helpful to write about?  **ASB_7EM_2M_G2_10**  It sounds like you have found some ways to manage your pain in the last day without opioid pain medication. Take some time to review how you have accomplished this. What has been working to reduce your pain? What strategies can you use to continue incorporating those helpful tools to reduce your pain to a lower level than it was today?  **ASB_7EM_2M_G2_11**  It seems that you have had some pain over the last day, but have not taken any opioid pain medications. Sometimes our personal self-image can have a big impact on how we feel. A quick and proven way to improve our own feelings of self-worth is to perform an act of kindness. What is something nice that you could do today for a friend, someone you love, or even a complete stranger? After you do it, notice how it makes you feel, not just emotionally, but physically.  **ASB_7EM_2M_G2_12**  It sounds like you have noticeable pain right now, but you may have also found ways to manage it. One technique for pain management is the tried and true, ice and heat methods. In general, use ice to treat acute injury or acute pain. Use heat to treat muscle or joint stiffness and chronic pain but not bruises, swellings, or open wounds.  [After an extended message is played GO TO SET 3: Extended Summary] |
| **Group 3: HIGH PAIN (> 6), NO OPIOID USE**  **ASB_7EM_2M_G3_01**  It sounds like you have been experiencing strong pain in the last day and did not take any opioid pain medication. Although it may not reduce pain completely, some people find it helpful to use mindful breathing to manage pain and the stress that comes with it. Here is one strategy for mindful breathing. Try placing a hand on your chest and one on your belly. Try to focus on breathing deeply into your stomach and avoiding any movement in your shoulders. Feel your stomach rise and fall. This could clear your mind from concentrating on the pain.  **ASB_7EM_2M_G3_02**  It sounds like you’ve been in quite a bit of pain over the last day, but haven’t taken any opioid pain medication. If you have a regular doctor, speaking with a nurse at your doctor’s office may be helpful in getting additional tips on managing severe pain. You could talk with the nurse about other ways to manage your pain without using opioid medications and concerns or questions you have about any medications. Just being able to discuss your pain with someone and feeling heard could help to reduce your stress around your pain.  **ASB_7EM_2M_G3_03**  It sounds like you have been in quite a bit of pain in the past day and haven’t used opioid pain medications. A lot of people have a belief that exercise can only make pain worse. Even though there may be some muscle pain for a short period of time, regular exercise can help keep you pain lower over time. Try taking a short walk around the block or inside the house. Building up to exercising 20 minutes a day has been shown to have many benefits including reducing pain. If you already meet this goal regularly, adding in more stretching, strength training, or another goal might be helpful.  **ASB_7EM_2M_G3_04**  It sounds like you have been dealing with some intense pain within the last day but didn’t take any opioid pain medications. Some people find relaxation helpful in managing pain. One method is to close your eyes and concentrate on relaxing your whole body by squeezing and relaxing one body part at a time. Try squeezing your right fist as hard as you can and relaxing it after five seconds. Then your left fist. Then your feet, one at a time, moving from one body part to the next all the way up to your head. Once you have done this to your entire body, lay there and feel the sense of relaxation for a few minutes.  **ASB_7EM_2M_G3_05**  It seems you have had strong pain in the last day and have not taken any opioid pain medications. A big part of pain management is getting enough good sleep. Think about how well you slept last night. Now think about what it takes for you to have a good night’s sleep. What is your routine you follow regularly before bed? Does it work to help you get good sleep?  **ASB_7EM_2M_G3_06**  It sounds like you are experiencing quite a bit of pain and haven’t used opioid pain medication. Overcoming the pain you are experiencing isn’t always easy to do. When people experience pain day in and day out, they can get a negative attitude about it, and understandably become irritable or feel frustrated easily. Trying to keep positive thoughts about your pain can be really helpful when facing your pain each day. Try thinking of four or five positive statements about things that you like about yourself, your life, or that make you happy. [pause] You could remind youself of these things to help keep yourself motivated when you experience pain.  **ASB_7EM_2M_G3_07**  It sounds like you’ve been having significant pain but you didn’t take any opioid pain medications in the last day. It could be helpful to think about things you’ve been doing over the last couple of days that may have given you relief from pain, even if only for a brief time. One strategy that other people have found helpful includes pacing how much they do at one time so that they don’t push themselves too hard. It is sometimes easier on your body to take several small breaks during your daily activities instead of pushing through, only to rest after. You may find your body is quicker to recover and you will experience less pain if you take several short breaks.  **ASB_7EM_2M_G3_08**  You have been managing your pain without taking pain medication. Managing pain can be difficult but you don’t have to do it alone. You may want to think about how you manage your pain, and consider sharing your methods with your doctor.Your doctor and you can work together to think through other pain management options or maintain what you are already doing. When you share your pain management plan with others, it can help you stay accountable to your goal.  **ASB_7EM_2M_G3_09**  It sounds like you have had strong pain in the last day and haven’t used opioid pain medications. One strategy that others who experience pain have found useful is tracking their pain levels compared to the activities they did that day. Writing down how you feel after a certain task can help you see patterns about how the task affects your pain. You don’t want to overwhelm your body with activities that tend to be followed by a lot of pain all on the same day.  **ASB_7EM_2M_G3_10**  It sounds like you have found some ways to manage your pain in the last day without opioid pain medication. You may want to take some time to review how you have accomplished managing your pain and getting through the day. What things seemed to help or reduce your pain, even if only for a short period of time?  **ASB_7EM_2M_G3_11**  It seems that you have had some significant pain over the last day. Sometimes our personal self-image can have a big impact on how we feel. A quick and proven way to improve our own feelings of self-worth is to perform an act of kindness. What is something nice you could do today for a friend, someone you love, or even a complete stranger? After you do it, notice how it makes you feel, not just emotionally, but physically.  **ASB_7EM_2M_G3_12**  It sounds like you have had strong pain over the last day and have not used opioid pain medications. One technique for pain management is ice and heat. When using ice, you want to make sure you are treating acute injury or acute pain. For heat, treating muscle or joint stiffness and chronic pain is recommended. Avoid using heat to treat bruises, swellings, or open wounds. Limit the time ice or heat is on the affected area to 20 minutes at a time.  [After an extended message is played GO TO SET 3: Extended Summary] |
| **Group 4: LOW PAIN (< 3), MEDICAL OPIOID USE**  **ASB_7EM_2M_G4_01**  It sounds like your pain has been fairly low and under control. Some people find it helpful to use mindful breathing in managing pain. Try placing a hand on your chest and one on your belly. Try to focus on breathing deeply into your stomach and avoiding any movement in your shoulders. Feel your stomach rise and fall. This could reduce your stress and clear your mind from concentrating on the pain.  **ASB_7EM_2M_G4_02**  It sounds like you have been experiencing a lower level of pain within the last day while taking your opioid pain medications. Asking for support from others is one option people have used to meet their physical and emotional needs when dealing with pain. Who are one or two people in your life that has been supportive to you in the past? Consider reaching out to them if your pain or stress worsens. Having someone to talk to can help reduce your anxiety.  **ASB_7EM_2M_G4_03**  It sounds like your pain has been under control. A lot of people have a mistaken belief that exercise can only make pain worse. Even though there may be a little discomfort for a short period of time, regular exercise can help prevent pain and speed up recovery in the future. It doesn’t have to be a lot, and can be something like taking a short walk around the block or inside the house. If you are recovering from an injury impacting one area of your body, consider excersises that could use other parts of your body. Building up to exercising 20 minutes a day has been shown to have many benefits including reducing pain. If you already meet this goal regularly, what are other goals that you might want for yourself, like strength training or being more flexible, that you could work towards?  **ASB_7EM_2M_G4_04**  It sounds like your pain has been under control lately. Some people find relaxation helpful in managing pain. One method is to close your eyes and concentrate on relaxing your whole body by squeezing and relaxing one body part at a time. Try squeezing your right fist as hard as you can and relaxing it after five seconds. Then your left fist. Then your feet, one at a time, moving from one body part to the next on your way to your head. Once you have done this to your entire body, allow yourself to be still and feel the sense of relaxation for a few minutes.  **ASB_7EM_2M_G4_05**  It seems you have had success in managing your pain in the last day while taking your opioid pain medication. A big part of pain management is getting enough good sleep. Think about how well you slept last night. What might have affected your sleep? What might be beneficial in the future to improve your sleep each night? Having a routine before bed has helped others feel more relaxed and prepared for sleep.  **ASB_7EM_2M_G4_06**  It sounds like your pain has been under control and you’ve taken some medications as part of your pain management. Some people have found that purposefully celebrating or enjoying the positive elements of their lives to be helpful when managing pain. What are some things for you that are positive in your life right now? Take some time for yourself to celebrate your life, work, family, friends or loved ones. Stop and enjoy a good meal, or a kind “hello”.  **ASB_7EM_2M_G4_07**  It sounds like your pain has been fairly low and under control. It could be helpful to think about things you’ve been doing over the last couple of days that helped you avoid having a lot of pain. One strategy that other people have found helpful is pacing how much they do at one time so that they don’t push themselves too hard. It is sometimes easier on your body to take several small breaks during your daily activities instead of pushing through only to rest at the end. You may find your body is quicker to recover and you experience less pain.  **ASB_7EM_2M_G4_08**  It sounds like your pain has been under control, and you’ve been using opioids in the way that they are prescribed. Following the doctor’s instructions for your medications is important to staying safe and healthy, and you have been successfully doing that over the last day.  **ASB_7EM_2M_G4_09**  It sounds like your pain has been under control, and you’ve been using opioids in the way that they were prescribed. In addition to the medication, some people find it helpful to write about their daily thoughts and activities in a journal. By writing things down, we can often find ourselves more at peace with our difficulties, feeling more grounded, or notice how our pain affects us. This might be something useful to continue keeping your pain level low. What are some things you could write about today?  **ASB_7EM_2M_G4_10**  It sounds like your pain has been under control, and you’ve been using opioids in the way that they are prescribed. Take some time to review how you have managed your pain this last day. Take note of strategies you may have used to avoid higher amounts of pain, in addition to your medications. What are those strategies? Think about ways you can incorporate these strategies to reduce your pain in the future.  **ASB_7EM_2M_G4_11**  You seem to be doing a great job of managing your pain over the last day while taking your opioid pain medications the way that they were prescribed to you. Sometimes our personal self-image can have a big impact on how we feel. A quick and proven way to improve our own feelings of self-worth is to perform an act of kindness. Try to do something nice today for a friend, someone you love, or even a complete stranger. Note how it makes you feel, not just emotionally, but physically.  **ASB_7EM_2M_G4_12**  It sounds like you have done a great job over the last day in managing your pain. One technique for pain management is the use of ice and heat. Many people use ice to treat acute injury or acute pain and heat to treat muscle or joint stiffness. You don’t want to use heat for bruises, swelling, or open wounds. Applying ice or heat for 20 minutes at a time can distract you from your pain and help reduce it.  [After an extended message is played GO TO SET 3: Extended Summary] |
| **Group 5: LOW PAIN (< 3), MEDICAL OPIOID MISUSE**  **ASB_7EM_2M_G5_01**  It sounds like your pain has been fairly low, and you have been taking your opioid pain medication differently than prescribed. Some people find it helpful to use mindful breathing to manage their pain or stress in their lives. Try placing a hand on your chest and one on your belly. Try to focus on breathing deeply into your stomach and avoiding any movement in your shoulders. Feel your stomach rise and fall. This could reduce your stress and clear your mind.  **ASB_7EM_2M_G5_02**  It sounds like you have been experiencing a lower level of pain within the last day while taking your opioid pain medications differently than prescribed. Asking for support from others is another way to have your physical and emotional needs met. Who are one or two people in your life that have been supportive to you? Consider reaching out to them to talk.  **ASB_7EM_2M_G5_03**  It sounds like your pain has been reasonably under control in the last day and you have been taking your opioid pain medication differently than prescribed. Even though there may be a little discomfort for a short period of time, regular exercise can help prevent pain and speed up recovery in the future. It doesn’t have to be a lot, and can be something like taking a short walk around the block or inside the house. Building up to exercising 20 minutes a day has been shown to have many benefits including reducing pain. If you already meet this goal regularly, what are other goals that you might want for yourself, like gaining strength or being more flexible, that you could be working towards?  **ASB_7EM_2M_G5_04**  It sounds like your pain has been reasonably under control and you have been taking your opioid prescription differently than prescribed in the last day. Some people find relaxation helpful in managing pain. One method is to close your eyes and concentrate on relaxing your whole body by squeezing and relaxing one body part at a time. Try squeezing your right fist as hard as you can and relaxing it after five seconds. Then your left fist. Then your feet, one at a time, moving from one body part to the next on your way up to your head. Once you have done this to your entire body, allow yourself to be still and feel the sense of relaxation for a few minutes.  **ASB_7EM_2M_G5_05**  It seems you have had success in managing your pain in the last day and you are taking your opioid pain medications differently than prescribed. A big part of preventing and managing pain is getting enough good sleep, but pain can also interfere with sleep, causing a cycle. Think about how well you slept last night. Now think about what it takes for you to have a good night’s sleep. What is the routine you regularly follow before bed? [pause] Having a night time routine which might include relaxation, stretching, or heat or ice right before bed may improve your sleep.  **ASB_7EM_2M_G5_06**  It sounds like your pain has been reasonably under control and you have been taking your opioid prescription differently than prescribed. Some people have found that purposefully celebrating or enjoying the positive elements of their lives to be effective at helping them manage their pain. What are some things for you that are positive in your life right now? Consider taking some time for yourself to celebrate your life, work, family, friends or loved ones. Stop and enjoy a good meal, or a kind “hello”.  **ASB_7EM_2M_G5_07**  It sounds like your pain has been mostly under control and you have been taking your opioid pain medication differently than prescribed. One strategy that other people have found helpful to keeping their pain low is pacing how much they do at one time so that they don’t push themselves too hard. It is sometimes easier on your body to take several small breaks during your daily activities instead of pushing through only to rest at the end. You may find your body is quicker to recover and you experience less pain if you take short breaks regularly.  **ASB_7EM_2M_G5_08**  It sounds like your pain has been reasonably well controlled, while taking your opioids outside of the recommended prescription. Following the doctor’s instructions is important to staying safe and healthy. Having a discussion with a healthcare professional about any pain you are experiencing especially pain that impacts taking your medications in a way that is different than prescribed could be important. It could help you adjust your plan for managing your pain to better meet your needs and keep your risks related to medication use lower overall.  **ASB_7EM_2M_G5_09**  It sounds like your pain has been mostly under control, and you’ve been using opioids differently than prescribed. Some people find it helpful to write about their daily thoughts and activities in a journal. By writing things down, we can often find ourselves more at peace with our difficulties, feel more grounded, or notice how our pain affects us. This might be something useful to continue keeping your pain level low. What would you write about today?  **ASB_7EM_2M_G5_10**  It sounds like your pain has been pretty well under control in the last day, and you’ve been using opioids differently than were prescribed. Think back to a time when you were experiencing pain and were able to handle it without using more than the amount of medication you were prescribed at that time. What were some of the strategies that you used at that time to help manage your pain? (Pause) How could you incorporate those options into your routine to continue to help with your pain and keep yourself safe at the same time?  **ASB_7EM_2M_G5_11**  You seem to be managing your pain over the last day while taking your opioid pain medications differently than prescribed. Sometimes our personal self-image can have a big impact on how we feel. A quick and proven way to improve our own feelings of self-worth is to perform an act of kindness. Try to do something nice today for a friend, someone you love, or even a complete stranger. Note how it makes you feel, not just emotionally, but physically.  **ASB_7EM_2M_G5_12**  It sounds like you have done well over the last day in managing your pain, and you have been taking your opioid pain medication differently than prescribed. One technique for pain management is the use of ice and heat. Many people use ice to treat acute injury or acute pain and heat to treat muscle or joint stiffness. You don’t want to use heat for bruises, swelling, or open wounds. Applying ice or heat for 20 minutes at a time can distract you from your pain and help reduce it.  [After an extended message is played GO TO SET 3: Extended Summary] |
| **Group 6: LOW PAIN (< 3), NON-MEDICAL OPIOID MISUSE**  **ASB_7EM_2M_G6_01**  It sounds like you have been experiencing a lower level of pain in the last day and you have been taking your opioid pain medications for reasons other than pain. Some people find it helpful to use mindful breathing to manage pain and the stress that comes with it. Try placing a hand on your chest and one on your belly. Try to focus on breathing deeply into your stomach and avoiding any movement in your shoulders. Feel your stomach rise and fall. This could clear your mind.  **ASB_7EM_2M_G6_02**  It sounds like you have been experiencing a lower level of pain within the last day and have been taking your opioid pain medications for reasons other than pain. Asking for support from others is another way to have your physical and emotional needs met. Who is someone in your life that has been supportive to you? Consider reaching out to that person to talk.  **ASB_7EM_2M_G6_03**  It sounds like your pain has been low and you have been taking opioid pain medications for reasons other than pain. A lot of people have a mistaken belief that exercise can only make pain worse. Even though there may be a little discomfort for a short period of time, regular exercise can help prevent pain and speed up recovery in the future. It doesn’t have to be a lot, and can be something like taking a short walk around the block or inside the house. Building up to exercising 20 minutes a day has been shown to have many benefits including reducing pain. If you already meet this goal regularly, what are other goals that you might want for yourself, like gaining strength or being more flexible, that you could work towards?  **ASB_7EM_2M_G6_04**  It sounds like you have been experiencing a lower level of pain within the last day and you have been taking your opioid pain medications for reasons other than pain. You may wish to consider a relaxation exercise the next time you have pain, stress, or difficulty sleeping. One relaxation exercise is to close your eyes and concentrate on relaxing your whole body by squeezing and relaxing one body part at a time. Try squeezing your right fist as hard as you can and relaxing it after five seconds. Then your left fist. Then your foot, one at a time, moving from one body part to the next on your way to your head. Once you have done this to your entire body, lay there and feel the sense of relaxation for a few minutes.  **ASB_7EM_2M_G6_05**  It seems you have had success in managing your pain in the last day and have taken your opioid pain medications for reasons other than pain. A big part of health is getting enough good sleep. Think about how well you slept last night. [pause] Now think about what it takes for you to have a good night’s sleep. [pause] What is the routine you regularly follow before bed? [pause] Getting into a night time routine which might include relaxation or stretching may improve your sleep.  **ASB_7EM_2M_G6_06**  It sounds like you have been able to reduce the pain you had in the past and you are taking opioids for reasons other than pain. Some people have found that purposefully celebrating or enjoying the positive elements of their lives to be helpful. Consider taking some time for yourself to celebrate your life, work, family, friends or loved ones. Stop and enjoy a good meal, a fun activity, or a kind “hello”.  **ASB_7EM_2M_G6_07**  It sounds like you don’t have much pain right now and you are using opioids for things other than pain. It could be helpful to think about things you’ve been doing over the last couple of days that helped you avoid experiencing a lot of pain or stress in your life aside from using opioids. One strategy that other people have found helpful is limiting how much they do at one time so that they don’t push themselves too hard. It is sometimes easier on your body and mind to take several small breaks during your daily activities instead of pushing through only to rest at the end.  **ASB_7EM_2M_G6_08**  It sounds like your pain is fairly well managed right now, and you are using opioids for issues other than pain. In addition to pain, some people experience anxiety, mood issues, inability to manage stress, or have difficulty sleeping. Talking with a doctor about the options available for you could help with reducing your pain further and managing your other symptoms.  **ASB_7EM_2M_G6_09**  It sounds like you don’t have much pain right now and you have been taking opioids for reasons other than pain. Writing down your thoughts, feelings, and activities can help relieve stress which can be related to reasons why you are taking opioids today. By writing things down, we often find ourselves more at peace with our difficulties, feel more grounded, or see patterns we hadn’t noticed. If you don’t already have one, perhaps you could start a journal today. What kinds of things would be helpful to write about?  **ASB_7EM_2M_G6_10**  It sounds like your pain is being managed right now, and you are using your opioids for things other than pain. It’s useful to recognize some of your body’s signs that the medication might be too much. You might notice that you are having trouble with constipation, nausea, dizziness, feeling out of it, sleepy, or difficulty breathing. These signs may be useful as you think about whether or not you want to try to cut back. Having a prescription for Naloxone, an opioid overdose reversal medication, could help reduce your risk. Consider talking to your doctor or a pharmacist about your risks and options for getting naloxone.  **ASB_7EM_2M_G6_11**  You seem to be doing a great job of managing your pain over the last day, and have taken your opioid pain medications for reasons other than pain. Sometimes our personal self-image can have a big impact on how we feel. A quick and proven way to improve our own feelings of self-worth is to perform an act of kindness. Try to do something nice today for a friend, someone you love, or even a complete stranger. Note how it makes you feel, not just emotionally, but physically.  **ASB_7EM_2M_G6_12**  It sounds like you don’t have much pain right now and you are using opioids for things other than pain. Pain can come and go. If you were to have pain again, one technique for pain management is ice and heat methods. In general, use ice to treat acute injury or acute pain. On the other hand, use heat to treat muscle or joint stiffness and chronic pain, but not bruises, swellings, or open wounds.  [After an extended message is played GO TO SET 3: Extended Summary] |
| **Group 7: MODERATE OR HIGH PAIN (> 3), MEDICAL OPIOID USE**  **ASB_7EM_2M_G7_01**  It sounds like you have been experiencing some significant pain in the last day while taking your prescribed opioid pain medications. Some people find it helpful to use mindful breathing to manage pain and the stress that comes with it. Try placing a hand on your chest and one on your belly. Try to focus on breathing deeply into your stomach and avoiding any movement in your shoulders. Feel your stomach rise and fall. This can help clear your mind from concentrating on your pain.  **ASB_7EM_2M_G7_02**  It sounds like you’ve had some significant pain over the past day, and you’ve been using your opioids as prescribed. You have been persistent at taking your medications safely even though your pain continues to bother you. Some people find it helpful to talk to their doctor, or find a doctor if they do not have one, when they continue to have strong pain despite treatment. A doctor or nurse could provide alternative ways to manage your pain and make it easier to stay within the amount you are prescribed when your medication doesn’t feel like it is fully covering you. Keeping track of where the pain is, how bad it is, what you have been doing to manage the pain, and what makes it better or worse could be helpful when discussing your pain with your doctor.  **ASB_7EM_2M_G7_03**  It sounds like you have had significant pain in the last day while taking your prescribed opioid medications. A lot of people have a mistaken belief that exercise can only make pain worse. Even though there may be a little discomfort for a short period of time, regular exercise can help keep your pain lower over time. Try taking a short walk around the block or inside the house. If your pain or an injury limits you, consider exercise that uses parts of your body not affected by pain today. Building up to exercising 20 minutes a day has been shown to have many benefits including reducing pain. If you already meet this goal regularly, what are other goals that you might want for yourself, like gaining strength or being more flexible, that you could work towards today?  **ASB_7EM_2M_G7_04**  It sounds like you have been dealing with some pain within the last day while taking your prescribed opioid pain medications. Some people find relaxation helpful in managing pain. One method is to close your eyes and concentrate on relaxing your whole body by squeezing and relaxing one body part at a time. Try squeezing your right fist as hard as you can and relaxing it after five seconds. Then your left fist. Then your feet, one at a time, moving from one body part to the next on your way to your head. Once you have done this to your entire body, lay there and feel the sense of relaxation for a few minutes.  **ASB_7EM_2M_G7_05**  It seems you’ve had significant pain in the last day and have taken your opioid pain medication as prescribed. A big part of pain management is getting enough good sleep, but pain can also interfere with sleep, causing a cycle. Think about how well you slept last night. Now think about what it takes for you to have a good night’s sleep. What’s the routine you regularly follow before bed? Having a night time routine which might include relaxation, stretching, or heat and ice right before bed may improve your sleep.  **ASB_7EM_2M_G7_06**  It sounds like you are experiencing significant pain and opioid medications have been one of the things you’ve used to manage it. Overcoming the pain you are experiencing isn’t always easy. When people experience pain day in and day out, they can get a negative attitude about it, and understandably feel frustrated. Concentrating on keeping your thoughts positive can be helpful when facing your pain and other challenges each day. Try thinking of five positive statements about what you are good at and what things about your life you enjoy. You could consider these statements when trying to keep your outlook positive.  **ASB_7EM_2M_G7_07**  It sounds like you are experiencing quite a bit of pain and you’ve been managing it with your prescribed opioid medication. It could be helpful to think about things you’ve been doing over the last couple of days that helped you avoid having more pain than you have now. One strategy that other people have found helpful is limiting how much they do at one time so that they don’t push themselves too hard. It is sometimes easier on your body to pace yourself by taking several small breaks during your daily activities instead of pushing through only to rest at the end. You may find your body is quicker to recover and you will experience less pain if you take several short breaks.  **ASB_7EM_2M_G7_08**  It sounds like you had strong pain today, and you stayed within the amount of medication that is prescribed to you. Sometimes the pain can be unbearable even with medication. Following the doctor’s instructions for your medications is important to staying safe and healthy, and you were able to do this over the last day.  **ASB_7EM_2M_G7_09**  It sounds like pain has been bothering you, and you’ve been managing it with your opioid pain medications. Some people find it helpful to write about their daily thoughts and activities in a journal. By writing things down we can often find ourselves at more peace with our difficulties, and feel more grounded and collected. Being able to recognize what activities cause you more pain can be beneficial to reducing your pain in the future.  **ASB_7EM_2M_G7_10**  It sounds like you have been bothered by pain in the last day while taking your prescribed opioid pain medication. You may want to take note of what you have done to avoid having your pain worsen, and ways you can continue using these tools each day to decrease the pain you experience.  **ASB_7EM_2M_G7_11**  It seems that you have had some pain over the last day, and have taken your opioid pain medications as prescribed. Sometimes our personal self-image can have a big impact on how we feel. A quick and proven way to improve our own feelings of self-worth is to perform an act of kindness. Try to do something nice today for a friend, someone you love, or even a complete stranger. Note how it makes you feel, not just emotionally, but physically.  **ASB_7EM_2M_G7_12**  It sounds like you have had some significant pain over the last day. One technique for pain management is the use of ice and heat. Many people use ice to treat acute injury or acute pain and heat to treat muscle or joint stiffness. Avoid using heat for bruises, swelling, or open wounds. Applying ice and heat for 20 minutes at a time can distract you from your pain and help reduce it.  [After an extended message is played GO TO SET 3: Extended Summary] |
| **Group 8: MODERATE OR HIGH PAIN (> 3), MEDICAL OPIOID MISUSE**  **ASB_7EM_2M_G8_01**  It sounds like you have been experiencing a lot of pain in the last day while taking your opioid pain medications differently than prescribed. Some people find it helpful to use mindful breathing to manage pain and the stress that comes with it. Try placing a hand on your chest and one on your belly. Try to focus on breathing deeply into your stomach and avoiding any movement in your shoulders. Feel your stomach rise and fall. This could clear your mind from always concentrating on your pain.  **ASB_7EM_2M_G8_02**  It sounds like you have been experiencing a lot of pain in the last day and have been taking your opioid pain medications differently than prescribed. Asking for support from others is another way to have your physical and emotional needs met so your pain doesn’t get worse. Who is someone in your life that has been supportive? Consider reaching out to them to talk about your pain. Having someone to talk to about your pain and the emotions surrounding it can help reduce your pain and stress.  **ASB_7EM_2M_G8_03**  It sounds like you have found some ways to manage your significant pain in the last day and have been taking your opioid medications differently than prescribed. A lot of people have a mistaken belief that exercise can only make pain worse. Even though there may be a little discomfort for a short period of time, regular exercise can help keep your pain lower over time. It can be as simple as taking a short walk around the block or inside the house. If your pain or an injury limits you, consider exercise that uses parts of your body not affected by pain today. Building up to exercising 20 minutes a day has been shown to have many benefits including reducing pain. If you already meet this goal regularly, what are other goals that you might want for yourself, like gaining strength or being more flexible, that you could work towards?  **ASB_7EM_2M_G8_04**  It sounds like you have been dealing with pain in the last day while taking your opioid pain medications differently than prescribed. Some people find relaxation helpful in managing pain. One method is to close your eyes and concentrate on relaxing your whole body by squeezing and relaxing one body part at a time. Try squeezing your right fist as hard as you can and relaxing it after five seconds. Then your left fist. Then your feet, one at a time, moving from one body part to the next on your way up to your head. Once you have done this to your entire body, lay there and feel the sense of relaxation for a few minutes.  **ASB_7EM_2M_G8_05**  It seems you’ve had significant pain in the last day and have taken your opioid pain medications differently than prescribed. A big part of pain management is getting enough good sleep, but pain can also interfere with sleep, causing a cycle. Think about how well you slept last night. Now think about what it takes for you to have a good night’s sleep. What’s the routine you regularly follow before bed? Getting into a night time routine which might include relaxation, stretching, or ice and heat right before bed may improve your sleep.  **ASB_7EM_2M_G8_06**  It sounds like you are experiencing significant pain and taking your opioid medications differently than prescribed. Overcoming the pain you are experiencing isn’t always easy to do. When people experience pain day in and day out, they can understandably feel frustrated. Trying to keep positive thoughts can be helpful when facing your pain and other challenges each day. Think of five positive statements about what you are good at, what is good about your life, or things that you enjoy. [pause] Consider saying these things to yourself to help keep your outlook positive.  **ASB_7EM_2M_G8_07**  It sounds like you are experiencing quite a bit of pain and you’ve been taking your opioid medications differently than prescribed. It could be helpful to think about things you’ve done in the past to reduce your pain in addition to your medication. One strategy that other people have found helpful is pacing how much they do at one time so that they don’t push themselves too hard. It is sometimes easier on your body to take several small breaks during your daily activities instead of pushing through only to rest at the end. You may find your body is quicker to recover and you will experience less pain if you take several short breaks.  **ASB_7EM_2M_G8_08**  It sounds like you’ve had strong pain today, and used opioid medication differently than prescribed. Following the doctor’s instructions is important to staying safe and healthy. Having a discussion with a healthcare professional could lead to an adjustment in your pain management plan to reduce your pain while keeping your risks related to medications low overall.  **ASB_7EM_2M_G8_09**  It sounds like pain has been bothering you quite a lot, and you’ve been taking your opioid medications differently than prescribed. Some people find it helpful to write about their daily thoughts and activities in a journal. By writing things down, we can often find ourselves more at peace with our difficulties, feel more grounded, or notice how our pain affects us. This might be something useful to you as you figure out how to best manage your pain. What kinds of things would it be helpful to write about?  **ASB_7EM_2M_G8_10**  It sounds like you have been having a lot of pain in the last day while taking your opioid pain medication differently than prescribed. Think back to a time when you were experiencing pain but were successful at using just your prescribed dose of medication. [pause] What were some of the strategies that you used at that time to help manage your pain? [pause] How could you incorporate those options into your routine to continue to help with your pain and keep your medication use consistent with how it was prescribed?  **ASB_7EM_2M_G8_11**  It seems that you have been bothered by pain over the last day, and have taken your opioid pain medications differently than prescribed. Sometimes our personal self-image can have a big impact on how we feel. A quick and proven way to improve our own feelings of self-worth is to perform an act of kindness. Try to do something nice today for a friend, someone you love, or even a complete stranger. Note how it makes you feel, not just emotionally, but physically.  **ASB_7EM_2M_G8_12**  It sounds like you have had some significant pain over the last day, and you have been taking your opioid medication differently than prescribed. One technique for pain management is the use of ice and heat. Many people use ice to treat acute injury or acute pain and heat to treat muscle or joint stiffness. You don’t want to use heat for bruises, swelling, or open wounds. Applying ice or heat for 20 minutes at a time can distract you from your pain and help reduce it.  [After an extended message is played GO TO SET 3: Extended Summary] |
| **Group 9: MODERATE OR HIGH PAIN (> 3), NON-MEDICAL OPIOID MISUSE**  **ASB_7EM_2M_G9_01**  It sounds like you have been experiencing some pain in the last day while taking opioids for reasons other than just pain. Some people find it helpful to use mindful breathing in managing pain. Try placing a hand on your chest and one on your belly. Try to focus on breathing deeply into your stomach and avoiding any movement in your shoulders. Feel your stomach rise and fall. This could reduce stress and clear your mind.  **ASB_7EM_2M_G9_02**  It sounds like you have been experiencing some significant pain within the last day. It also sounds like you use your opioid pain medications for reasons other than pain. Asking for support from others is another way to have your physical and emotional needs met so your pain does not get worse. Who is someone in your life that has been supportive? Consider reaching out to them to talk about your pain. Having someone to talk to about your pain and the emotions that surround it could be helpful in reducing your pain and stress.  **ASB_7EM_2M_G9_03**  It sounds like you are working on ways to manage your significant pain in the last day while taking opioid medication for reasons other than just pain. A lot of people have a mistaken belief that exercise can only make pain worse. Even though there may be a little discomfort for a short period of time, regular exercise can help keep your pain lower over time. In can be as simple as taking a short walk around the block or inside the house. If your pain or an injury limits you, consider exercise that uses parts of your body not affected by pain today. Building up to exercising 20 minutes a day has been shown to have many benefits including reducing pain. If you already meet this goal regularly, adding in more stretching or strength training, or another goal you might want for yourself, can be helpful.  **ASB_7EM_2M_G9_04**  It sounds like you have been dealing with some pain within the last day and you’ve been using your opioid medications to treat symptoms other than pain. Some people find relaxation helpful in managing pain or other things that bother them. One method is to close your eyes and concentrate on relaxing your whole body by squeezing and relaxing one body part at a time. Try squeezing your right fist as hard as you can and relaxing it after five seconds. Then your left fist. Then your feet, one at a time, moving from one body part to the next on your way to your head. Once you have done this to your entire body, lay there and feel the sense of relaxation for a few minutes.  **ASB_7EM_2M_G9_05**  It seems you have had significant pain in the last day and have taken opioids for reasons other than pain. A big part of pain management is getting enough good sleep, but pain can also interfere with sleep, causing a cycle. Think about how well you slept last night. [pause] Now think about what it takes for you to have a good night’s sleep. What is the routine you regularly follow before bed? [pause] Getting into a night time routine which might include relaxation, stretching, or heat and ice right before bed may improve your sleep.  **ASB_7EM_2M_G9_06**  It sounds like you are experiencing significant pain and you’ve been taking opioid medications to manage the pain, and other reasons. Overcoming the pain you are experiencing isn’t always easy to do. Some people have found that purposefully celebrating or enjoying the positive elements of their lives to be effective at helping them manage their pain. Consider taking some time to yourself to celebrate your life, work, family, friends or loved ones. Stop and enjoy a good meal, or a kind “hello”.  **ASB_7EM_2M_G9_07**  It sounds like you are experiencing quite a bit of pain and you’ve been managing it with opioid medication. You have also been using your opioid medications for reasons other than pain. One strategy that other people have found helpful to reduce their pain is limiting how much they do at one time so that they don’t push themselves too hard. It is sometimes easier on your body to take several small breaks and pace yourself during your daily activities instead of pushing through to the end. You may find your body is quicker to recover and you experience less pain if you take short breaks regularly.  **ASB_7EM_2M_G9_08**  It sounds like you’ve had some significant pain over the last day, and you’ve been using opioids to treat your pain, and other symptoms. Some people find it helpful to talk to their doctor, or find a doctor if they do not have one, when they are in this situation. Preventing yourself from taking more pain medication than prescribed is the healthiest thing you can do. A doctor could adjust your pain management plan and provide alternative ways to manage your pain when your medication doesn’t feel like it is fully covering you. Keeping track of where the pain is, how bad it is, what you have been doing to manage the pain, and what makes it better or worse could be helpful when discussing your pain with your doctor.  **ASB_7EM_2M_G9_09**  It sounds like pain has been bothering you, and you’ve been taking opioid medications for pain and for other reasons. Writing down our thoughts, feelings, and activities can help relieve stress which can be related to pain you might experience. By writing things down, we often find ourselves more at peace with our difficulties, feel more grounded, or see patterns we hadn’t noticed. If you don’t already have one, perhaps you could start a journal today. What kinds of things would be helpful to write about?  **ASB_7EM_2M_G9_10**  It sounds like you’ve been having significant pain in the last day and are taking opioid pain medications. You have also been using your opioid pain medication to treat symptoms other than pain. When experiencing a higher level of pain and using more medication than prescribed, it’s useful to recognize some of our body’s signs that the medication might be too much. You might notice that you are having trouble with constipation, nausea, dizziness, feeling out of it, sleepy, or difficulty breathing. These signs may be useful as you think about whether or not you want to try to cut back. Having a prescription for Naloxone, an opioid overdose reversal medication, could help reduce your risk. Consider talking to your doctor or pharmacist about your risks and naloxone options.  **ASB_7EM_2M_G9_11**  It seems that you have had some pain over the last day, and you are using your opioid pain medications to treat things other than pain. Sometimes our personal self-image can have a huge impact on how we feel. A quick and proven way to improve our own feelings of self-worth is to perform an act of kindness. You could try to do something nice today for a friend, someone you love, or even a complete stranger. Note how it makes you feel, not just emotionally, but physically.  **ASB_7EM_2M_G9_12**  It sounds like you have had some significant pain over the last day, and you’ve been using your opioid pain medication to treat symptoms other than pain. One technique for pain management is the use of ice and heat. Many people use ice to treat acute injury or acute pain and heat to treat muscle or joint stiffness. You don’t want to use heat for bruises, swelling, or open wounds. Applying ice or heat for 20 minutes at a time can distract you from your pain and help reduce it.  [After an extended message is played GO TO SET 3: Extended Summary] |

| **Set 3: Extended Summary** |
| --- |
| [Play an extended summary from below based on the Extended Message group the patient received.]  **ASB_7EM_3S_1Intro**  Thanks for all of your answers today.  **Group 1**  **ASB_7EM_3S_1Intro_G1**  You have told us that you have not had much pain, and have not been using opioid medications.  **Group 2 and Group 3**  **ASB_7EM_3S_1Intro_G2_G3**  You have told us that you have had pain, and you have not been using opioid medications.  **Group 4**  **ASB_7EM_3S_1Intro_G4**  You have told us that you have not had much pain, and you have been taking your prescription as prescribed.  **Group 5 and Group 6**  **ASB_7EM_3S_1Intro_G5_G6**  You have told us that you have not had much pain, and you have been taking your prescription differently than prescribed.  **Group 7**  **ASB_7EM_3S_1Intro_G7**  You have told us that you have had pain, and you have not been taking your prescription differently than prescribed.  **Group 8 and Group 9**  **ASB_7EM_3S_1Intro_G8_G9**  You have told us that you have had pain, and you have been taking your prescription differently than prescribed.  [IF the Extended Question was from the Behavioral Intentions group, THEN GO TO ASB_7EM_3S_2Behav]  [IF the Extended Question was from the Tools for Change group, THEN GO TO ASB_7EM_3S_3Tool]  [IF ELSE, GO TO Section: Conclusion] |
| **ASB_7EM_3S_2Behav**  [IF the extended question is from the Behavioral Intentions group include the appropriate Behavioral Intentions summary]  **ASB_7EM_3S_2Behav1a** (Behav1=0,1,2)  You have also told us that you feel not at all confident about your ability to manage your current pain.  **ASB_7EM_3S_2Behav1b** (Behav1=3,4,5,6)  You have also told us that you feel somewhat confident about your ability to manage your current pain.  **ASB_7EM_3S_2Behav1c** (Behav1=7,8,9,10)  You have also told us that you feel very confident about your ability to manage your current pain.  **ASB_7EM_3S_2Behav2a** (Behav2=0,1,2)  You have also told us that you feel that it is not at all important to you today to find ways to improve how you manage pain in the future.  **ASB_7EM_3S_2Behav2b** (Behav2=3,4,5,6)  You have also told us that you feel that it is somewhat important to you today to find ways to improve how you manage pain in the future.  **ASB_7EM_3S_2Behav2c** (Behav2=7,8,9,10)  You have also told us that you feel that it is very important to you today to find ways to improve how you manage your pain in the future.  **ASB_7EM_3S_2Behav3a** (Behav3=0,1,2)  You have also told us that you are not at all confident that you can use your opioids safely.  **ASB_7EM_3S_2Behav3b** (Behav3=3,4,5,6)  You have also told us that you are somewhat confident that you can use your opioids safely.  **ASB_7EM_3S_2Behav3c** (Behav3=7,8,9,10)  You have also told us that you are very confident that you can use your opioids safely.  **ASB_7EM_3S_2Behav4a** (Behav4=0,1,2)  Lastly, you have also told us that you feel that it is not at all likely that your use of opioids to manage your pain will be different in one month from now than it is today.  **ASB_7EM_3S_2Behav4b** (Behav4=3,4,5,6)  Lastly, you have also told us that you feel that it is somewhat likely that your use of opioids to manage your pain will be different in one month from now than it is today.  **ASB_7EM_3S_2Behav4c** (Behav4=7,8,9,10)  Lastly, you have also told us that you feel that it is very likely that your use of opioids to manage your pain will be different in one month from now than it is today.  [After a summary is played GO TO Section: Conclusion] |
| **ASB_7EM_3S_3Tool**  [IF the extended question is from the Tools for Change group include the appropriate Tools for Change summary]  **ASB_7EM_3S_3Tool1a** (Tool1=1)  You have also chosen speak with a physician or therapist to talk to my doctor about my pain or medication use as a tool to help you right now.  **ASB_7EM_3S_3Tool1b** (Tool1=2)  You have also chosen speak with a physician or therapist to discuss the side effects I experience from my medications as a tool to help you right now.  **ASB_7EM_3S_3Tool1c** (Tool1=3)  You have also chosen speak with a physician or therapist to recognize withdrawal symptoms while reducing or stopping opioid medication as a tool to help you right now.  **ASB_7EM_3S_3Tool1d** (Tool1=4)  You have also chosen speak with a physician or therapist to discuss ways to manage pain that I am not already using as a tool to help you right now.  **ASB_7EM_3S_3Tool1e (Tool1=5)**  You have also chosen speak with a physician or therapist about other topics as a tool to help you right now.  **ASB_7EM_3S_3Tool2a** (Tool2=1)  You have also chosen develop coping skills or alternative options for my pain and exercise, stretching or yoga as a tool to help you right now.  **ASB_7EM_3S_3Tool2b** (Tool2=2)  You have also chosen develop coping skills or alternative options for my pain and spend time with people who I find encouraging as a tool to help you right now.  **ASB_7EM_3S_3Tool2c** (Tool2=3)  You have also chosen develop coping skills or alternative options for my pain and take medication according to the instructions from my doctor as a tool to help you right now.  **ASB_7EM_3S_3Tool2d** (Tool2=4)  You have also chosen develop coping skills or alternative options for my pain and read, write in a journal, or watch television as a tool to help you right now.  **ASB_7EM_3S_3Tool2e** (Tool2=5)  You have chosen develop coping skills or alternative options for my pain and spend some time doing a hobby I enjoy, or trying a new one as a tool to help you right now.  **ASB_7EM_3S_3Tool2f** (Tool2=6)  You have also chosen develop coping skills or alternative options for my pain and trying something else to help manage my pain or other problems as a tool to help you right now.  **ASB_7EM_3S_3Tool3a** (Tool3=1)  You have chosen work to improve my health and start an exercise, stretching or yoga routine as a tool to help you right now.  **ASB_7EM_3S_3Tool3b** (Tool3=2)  You have chosen work to improve my health and reduce my stress level by meditating or deep breathing as a tool to help you right now.  **ASB_7EM_3S_3Tool3c** (Tool3=3)  You have chosen work to improve my health and change one health habit, like how much I drink or smoke as a tool to help you right now.  **ASB_7EM_3S_3Tool3d** (Tool3=4)  You have chosen work to improve my health and concentrate on eating healthier foods, less salt, or less fast food as a tool to help you right now.  **ASB_7EM_3S_3Tool3e** (Tool3=5)  You have chosen work to improve my health and drink less soda or eat less sugary foods as a tool to help you right now.  **ASB_7EM_3S_3Tool3f** (Tool3=6)  You have chosen work to improve my health and do things that help me connect with myself or my spiritual side as a tool to help you right now.  **ASB_7EM_3S_3Tool3g** (Tool3=7)  You have chosen work to improve my health and find enjoyable hobbies as a tool to help you right now.  **ASB_7EM_3S_3Tool3h** (Tool3=8)  You have chosen work to improve my health and making a change to something else as a tool to help you right now.  [After a summary is played GO TO Section: Conclusion] |

# Phone Session

| **ASB_8**  Thank you for your responses. A member of our staff will be calling you back soon to talk further about your symptoms.  [GO TO Section: Conclusion] |
| --- |

# Conclusion

| **ASB_9**  If you have a question for the study team, please call us at 734-232-0387.  [GO TO ENDCall] |
| --- |
