# Supplementary material for: An Automatically Adaptive Digital Health Intervention to Decrease Opioid-Related Risk While Conserving Counselor Time: Quantitative Analysis of Treatment Decisions Based on Artificial Intelligence and Patient-Reported Risk Measures
Source: J Med Internet Res. 2023 Jul 11;25:e44165. doi: 10.2196/44165 (PMC10369305; doi:10.2196/44165)
Supplement: Multimedia Appendix 3 [file jmir_v25i1e44165_app3.docx]

Description of Variables Used in RL Decision-Making

Each week, participants get feedback that is decided by an AI engine. This engine chooses one of (i) brief IVR message (ii) extended IVR message (iii) short therapist session for each participant each week. The AI engine uses a regression based algorithm known as LinUCB to make these decisions, and to do so it constructs a feature vector representing a summary of the participant’s interaction with the system. The components of this feature vector are described in the table below:

**Feature vector**

| **Feature** | **How measured** |
| --- | --- |
| **From Baseline Survey** |  |
| 1. Baseline COMM | 0-32 scale normalized to between 0-1 |
| 1. Baseline severity of pain | 0-10 scale normalized to between 0-1 |
| 1. Baseline number of substances used | 0-10 scale normalized to between 0-1 |
| **From Daily IVR Responses** |  |
| 1. Average of last week pain score | 0-10 scale normalized to between 0-1 |
| 1. Change in average pain score from week before | change between -100% to 100% normalized to between 0 -1 |
| 1. Average of last week pain interference scores | 0-10 scale normalized to between 0-1 |
| 1. Change in average pain interference score from week before | change between -100% to 100% normalized to between 0 -1 |
| 1. Avg of self-reported opioid use with recency bias | 0-10 scale normalized to between 0-1 |
| **Summary statistics** |  |
| 1. Summary of rewards received when brief messages are used for this patient (with recency bias) | This is a weighted average (more weight on recent sessions) of the rewards that the engine has received when giving brief messages to the patient. (normalized between 0-1) |
| 1. Summary of rewards received when extended messages are used for this patient (with recency bias) | This is a weighted average (more weight on recent sessions) of the rewards that the engine has received when giving extended messages to the patient. (normalized between 0-1) |
| 1. Summary of rewards received when therapist sessions are used for this patient (with recency bias) | This is a weighted average (more weight on recent sessions) of the rewards that the engine has received when giving therapist sessions to the patient. (normalized between 0-1) |
| **Time based** |  |
| 1. Week of intervention | Whether the current session is the 1^st^, 2^nd^,…12^th^  normalized between 0-1. |

When the AI engine is making a decision it uses weights learned by that time, for each of the features, and calculates an expected value for making each decision. The engine then chooses the decision for that patient with the highest expected value, unless there are multiple choices with expected values that are deemed “close enough”. The idea behind this “close enough” threshold is that if an IVR message is close enough in value to a therapist session, then the IVR session should be chosen in order to preserve the limited therapist time. In cases where both the brief and extended IVR choices are deemed close enough, a random choice between the two is made, since there is no therapist time required for either.

The engine then receives feedback from the weekly calls and calculates a reward based on the self-reported opioid use. This reward is then used to update the weights for the next decision making time.

The reward, r, is calculated from self reported opioid use during the daily calls and averaged for the week. The formula used in calculating the reward is

If ASB_2B2 = 0, *r = 6*

Else *r = (ASB_3Q1 - 1) +(ASB_3Q2 - 1) +(ASB_3Q3 - 1)*

Where

ASB_2B2 = Average of responses this week to “In the past 24 hours, how many opioid pills did you take? Please enter the number of pills. ”

ASB_3Q1 = Average of responses this week to “In the past 24hours, how often have you needed to take your opioid pain medication more often or at a greater amount than prescribed in order to relieve your pain? If several times, press 1. If once or twice, press 2. Or if not at all, press 3.”

ASB_3Q2 = Average of responses this week to “In the past 24 hours, how often have you taken opioid pain medication that belonged to friends, family, or someone else? If several times, press 1. If once or twice, press 2. Or if not at all, press 3.”

ASB_3Q3 = Average of responses this week to “In the past 24 hours, how often have you used your opioid pain medication to help with other symptoms, such as problems sleeping, being nervous or anxious, or feeling sad or stressed? If several times, press 1. If once or twice, press 2. Or if not at all, press 3.”
